# Activating a [FeFe] Hydrogenase Mimic for Hydrogen Evolution under Visible Light**

**DOI:** 10.1002/anie.202202079

**Published:** 2022-03-23

**Authors:** Philipp Buday, Chizuru Kasahara, Elisabeth Hofmeister, Daniel Kowalczyk, Micheal K. Farh, Saskia Riediger, Martin Schulz, Maria Wächtler, Shunsuke Furukawa, Masaichi Saito, Dirk Ziegenbalg, Stefanie Gräfe, Peter Bäuerle, Stephan Kupfer, Benjamin Dietzek‐Ivanšić, Wolfgang Weigand

**Affiliations:** ^1^ Institute of Inorganic and Analytical Chemistry Friedrich Schiller University Jena Humboldtstraße 8 07743 Jena Germany; ^2^ Department of Chemistry Graduate School of Science and Engineering Saitama University Shimo-okubo, Sakura-ku, Saitama City, Saitama 338-8570 Japan; ^3^ Department Functional Interfaces Leibniz Institute of Photonic Technology Jena (Leibniz-IPHT) Albert-Einstein-Straße 9 07745 Jena Germany; ^4^ Institute of Chemical Engineering Ulm University Albert-Einstein-Allee 11 89081 Ulm Germany; ^5^ Institute of Physical Chemistry Friedrich Schiller University Jena Helmholtzweg 4 07743 Jena Germany; ^6^ Abbe Center of Photonics (ACP) Friedrich Schiller University Jena Albert-Einstein-Straße 6 07745 Jena Germany; ^7^ Institute of Organic Chemistry II and Advanced Materials Ulm University Albert-Einstein-Allee 11 89081 Ulm Germany; ^8^ Center for Energy and Environmental Chemistry Jena (CEEC Jena) Friedrich Schiller University Jena Philosophenweg 8 07743 Jena Germany; ^9^ Fraunhofer Institute for Applied Optics and Precision Engineering Albert-Einstein-Straße 7 07745 Jena Germany

**Keywords:** H_2_ Production, Oligothiophene, *Operando* EPR Spectroscopy, Photocatalysis, [FeFe] Hydrogenase Mimics

## Abstract

Inspired by the active center of the natural [FeFe] hydrogenases, we designed a compact and precious metal‐free photosensitizer‐catalyst dyad (**PS‐CAT**) for photocatalytic hydrogen evolution under visible light irradiation. **PS‐CAT** represents a prototype dyad comprising π‐conjugated oligothiophenes as light absorbers. **PS‐CAT** and its interaction with the sacrificial donor 1,3‐dimethyl‐2‐phenylbenzimidazoline were studied by steady‐state and time‐resolved spectroscopy coupled with electrochemical techniques and visible light‐driven photocatalytic investigations. *Operando* EPR spectroscopy revealed the formation of an active [Fe^I^Fe^0^] species—in accordance with theoretical calculations—presumably driving photocatalysis effectively (TON≈210).

## Graphical abstract

A precious metal‐free photocatalyst **PS‐CAT** consisting of a [FeFe] hydrogenase mimic was utilized in the scope of hydrogen generation under visible light. The dyad comprising a π‐conjugated oligothiophene as light absorber shows a remarkably high photocatalytic activity. In accordance with theory, *operando* EPR spectroscopy using BIH as sacrificial donor reveals the generation of an active [Fe^I^Fe^0^] species during the irradiation experiment.

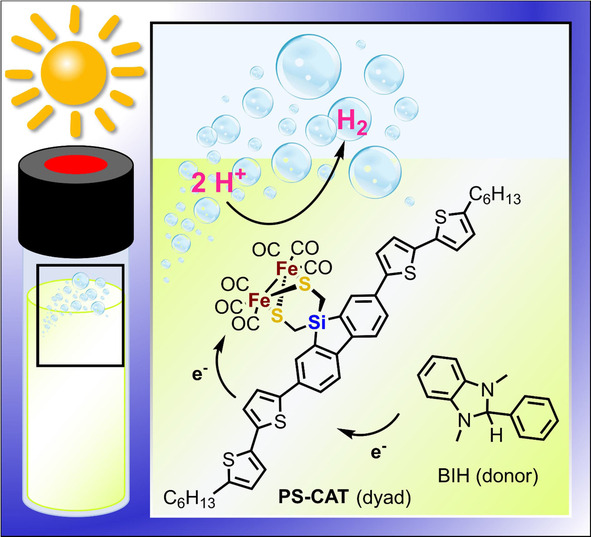

Nature provides paradigms for photocatalysis to generate solar fuels in a renewable and climate‐neutral fashion. In this context, scientists have created various architectures to perform catalytic key processes, e.g. the hydrogen evolution reaction (HER), under light excitation. Commonly, 4d and 5d transition metals (e.g. Ru, Cd, Re, Ir, Pt) play a key role in molecular artificial HER photocatalysis—both as light‐harvesters and catalysts—due to their favourable (photo)redox chemistry and stability.^[1]^ However, rare abundance or undesired side effects (e.g. toxicity) hamper a larger scale application.

Nature uses earth‐abundant, inexpensive reaction centers in the [FeFe] hydrogenase enzyme for HER.^[2]^ Inspired by the natural paradigm, [FeFe] hydrogenase mimics have been reported.^[3]^ Such catalysts, however, often rely on photosensitizers based on precious metals. Few exceptions are given by e.g. xanthene dyes, (zinc) porphyrins[^1a^, ^4^] or carbon dots^[5]^ as light harvesters. Recently, we reported another fully precious metal‐free example, a dibenzosilole photosensitizer directly attached to the catalyst, yield a TON of 539 for HER under UV light (254 nm).^[6]^ π‐Conjugated oligothiophenes present metal‐free, readily accessible and strong light absorbers in the visible spectral region. The optical and redox properties of such dyes, which are used e.g. in organic^[7]^ or dye‐sensitized^[8]^ solar cells, can be tuned easily. Recently, Hammarström and colleagues combined a π‐conjugated oligothiophene photosensitizer and a [FeFe] hydrogenase mimic in a cosensitized NiO photocathode.^[9]^

We report a fully precious metal‐free, visible‐light absorbing, bioinspired and compact photocatalyst dyad **PS‐CAT** as a prototype hybrid comprising π‐conjugated oligothiophenes as photosensitizer for the light‐driven HER. We present **PS‐CAT**, its photocatalytic activity in the presence of the sacrificial donor 1,3‐dimethyl‐2‐phenylbenzimidazoline (BIH) and a detailed mechanistic characterization. The oligothiophene sensitizer unit allows for a major improvement compared to the previously reported dibenzosilole light harvester, which is only active under UV irradiation.

**PS‐CAT** combines the light‐harvesting properties of π‐conjugated oligothiophenes (PS) with a bioinspired [FeFe] hydrogenase mimic (CAT). It was synthesized according to Scheme 1 (also detailed in Scheme S1) from the bis(thioacetate) **PS**. This pathway (iii–v) comprising a *N*‐Methyl‐2‐pyrrolidone (NMP)‐mediated^[10]^ complexation reaction in step v) resulted in a better overall yield of **PS‐CAT** (17 %) compared to a direct conjunction (vi) with the [2Fe2S] cluster (8 %). Both, **PS** and **PS‐CAT** were characterized by NMR, IR spectroscopy and mass spectrometry. The molecular structure of **PS‐CAT** (Figure 1; Figure S2 and Table S1/S2; Supporting Information for crystallographic details) reveals a similar binding mode compared to that of a previous reported, structurally related complex.^[6]^ A view on the ab plane shows stacking of planar **PS‐CAT** molecules (Figure S2b). The cyclic voltammogram of **PS‐CAT** in CH_2_Cl_2_ at a scan rate of 0.2 Vs^−1^ shows a quasi‐reversible reduction event at *E*_1/2_^Red1^=−1.61 V vs. Fc^+^/Fc (Figure S9a).

**Scheme 1:**
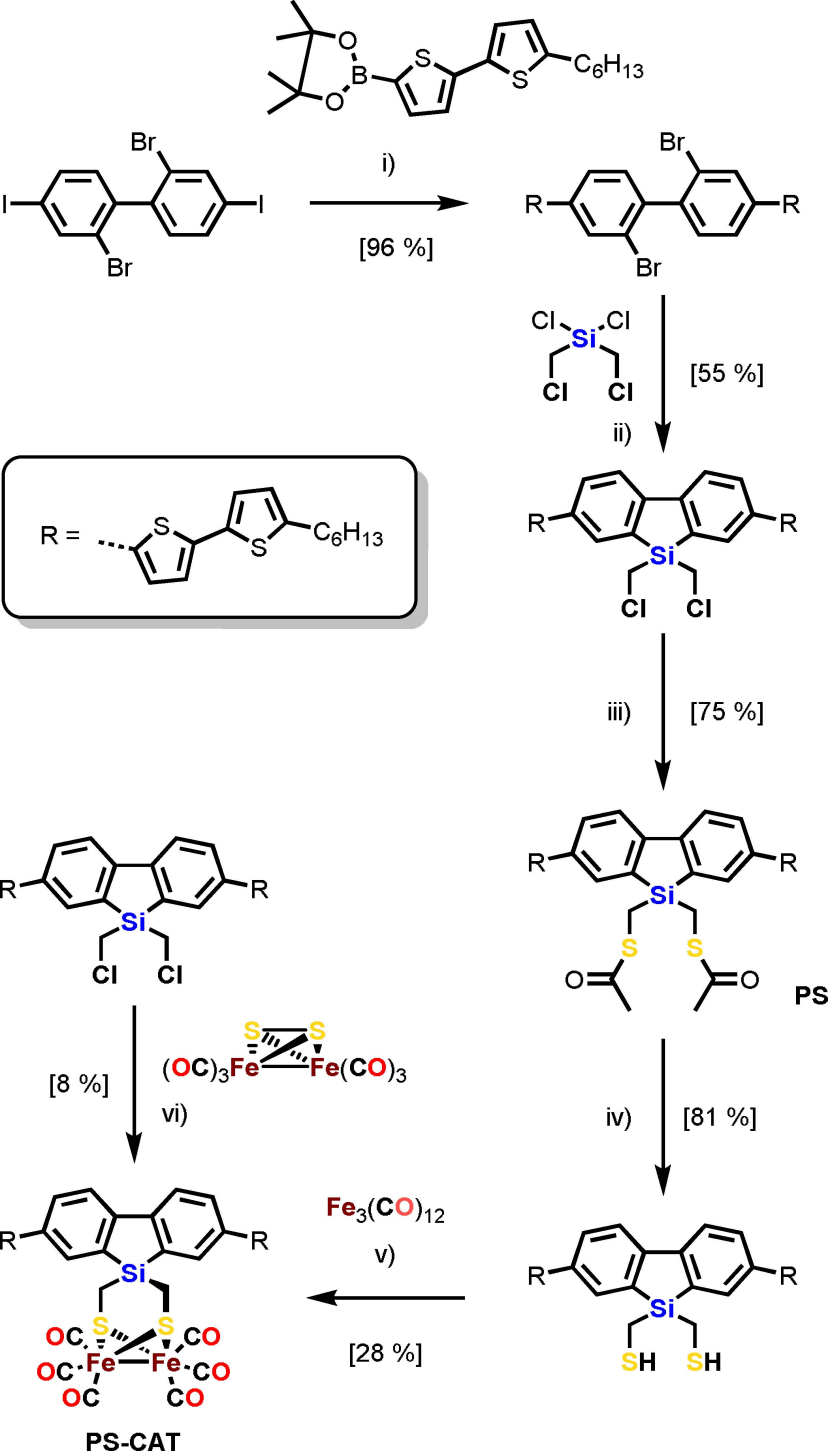
Synthetic pathway to **PS‐CAT** via **PS**. i) BnNEt_3_Cl, [Pd(PPh_3_)_4_], K_2_CO_3_, toluene; 60 °C, 12 h; ii) *n*‐BuLi, THF; −78 °C to r. t., 12 h; iii) KSCOCH_3_, THF; r. t., 20 h; iv) LiAlH_4_, Et_2_O; 0 °C to r. t., 12 h; v) Fe_3_(CO)_12_, toluene/NMP (20 : 1); r. t., 12 h; vi) LiBHEt_3_, THF, −90 °C to r. t., 14 h.

**Figure 1:**
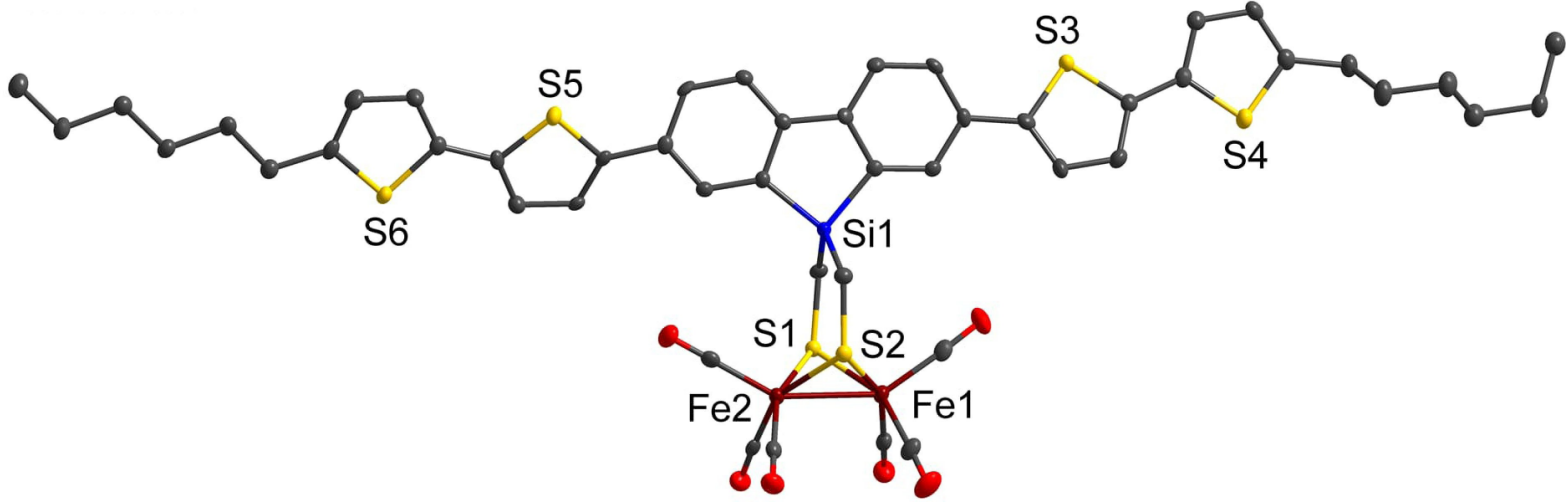
Molecular structure and atom labeling scheme of **PS‐CAT**.^[11]^ The ellipsoids represent a probability of 50 %. Hydrogen atoms are omitted for clarity. For selected bond lengths and angles see Table S2.

The photocatalytic proton reduction behaviour of **PS‐CAT** was studied under irradiation with visible light in a 5 : 1 mixture of CH_3_CN and NMP and with the two‐electron, one‐proton sacrificial donor BIH^[12]^ (1000 equivalents). The solvent mixture ensures solubility and a good catalytic performance due to an overall high dielectric constant. Moreover, the weak base NMP can potentially react with the oxidized donor (BIH^.+^) to capture the proton, thus inhibiting potential back electron transfer processes.^[13]^ The photocatalytic reactions were performed in a custom‐made, modular 3D printed photoreactor setup (Figure S1). Figure 2 shows the hydrogen evolution time profile. Within the initial 23 hours the catalytic turnover increases slightly sigmoidally, before the hydrogen evolution reaches a plateau at TON≈210 (TOF≈6.4 h^−1^). This visible light activity is the highest reported for molecular dyads employing [FeFe] hydrogenase mimics.[^1a^, ^4^, ^14^] No hydrogen was detected in corresponding experiments without **PS‐CAT**, BIH or both or in the dark. Further photocatalytic results are given in Table S3. The major advance of **PS‐CAT** over our previous reported photocatalyst dyad^[6]^ is its pronounced catalytic performance under visible light irradiation (455 nm LED) even under comparatively low intensities of 10 mW cm^−2^. Moreover, **PS‐CAT** shows a significantly prolonged catalytic turnover up to 33 hours (7 hours in our earlier work^[6c]^). This is likely a consequence of the lower energy light used here with respect to the previous study (UV light irradiation) and hence avoidance of side reactions. In the latter small amounts of hydrogen were detected in the absence of the dyad, which was not the case in this work.

**Figure 2:**
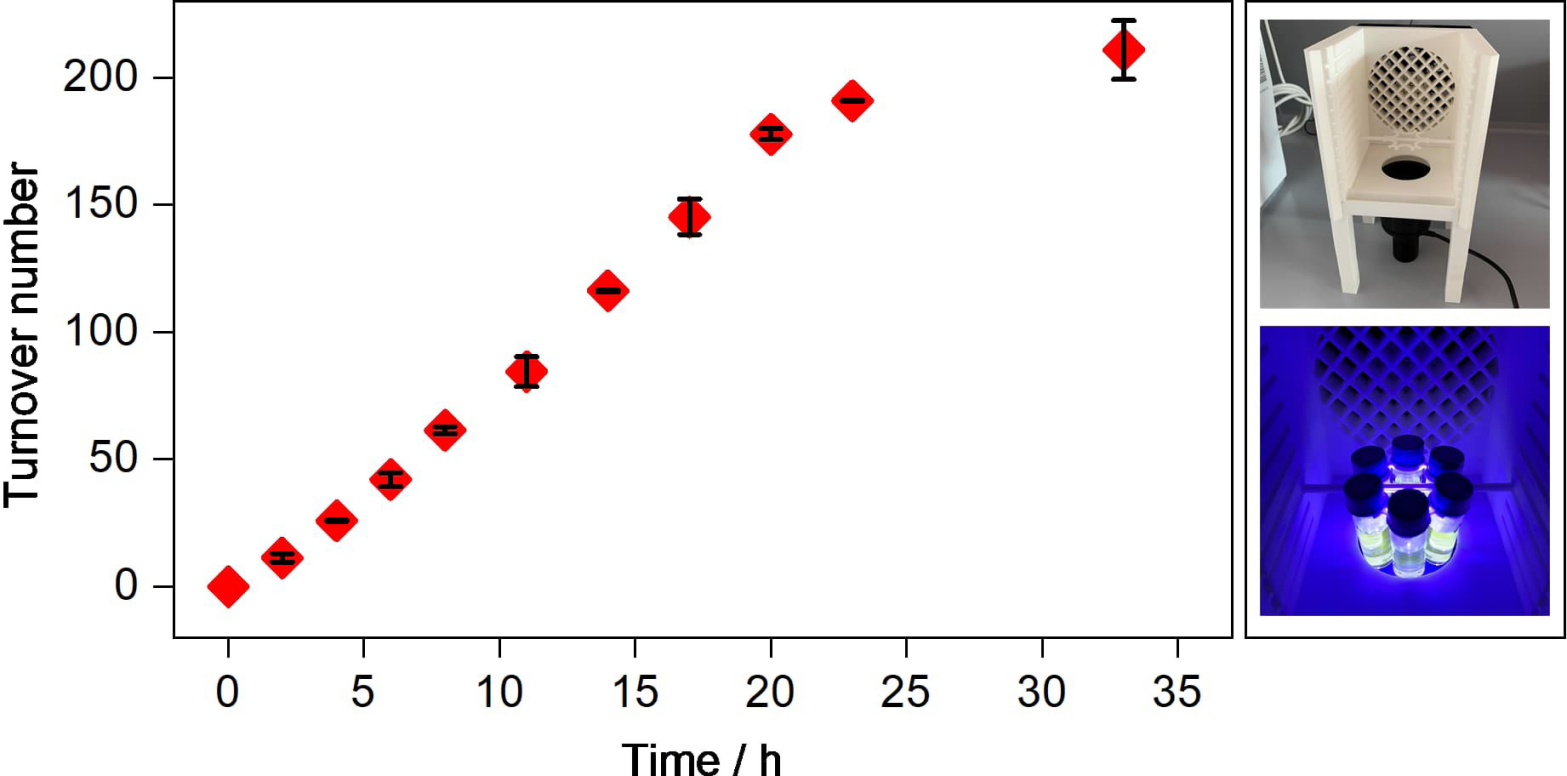
Photocatalytic hydrogen production by **PS‐CAT** (10 μM) in CH_3_CN/NMP (5 : 1) with BIH (1000 equivalents) upon irradiation at 455 nm in a 3D printed photoreactor platform (see pictures on the right side). Hydrogen was quantified by GC‐TCD with samples drawn from the head space. Each point was determined in duplicate and the error bars give the range of variation.

Figure 3a depicts the UV/Vis spectra of **PS** and **PS‐CAT**, which show a strong absorption in the visible spectral region (*λ*_max_=409 nm, *ϵ*≈8.1 ⋅ 10^4^ L mol^−1^ cm^−1^ for **PS** and *λ*_max_=410 nm, *ϵ*≈6.1 ⋅ 10^4^ L mol^−1^ cm^−1^ for **PS‐CAT**) resulting from a ππ* transition localized within the oligothiophene moiety (see S_1_ in Figure 3d). For **PS‐CAT** TDDFT reveals a dipole allowed charge‐transfer transition in the visible region (see S_2_ in Figure 3d) shifting electron density from a photosensitizer‐localized π orbital to the [FeFe] hydrogenase mimic unit, i.e. into the σ* orbital of the Fe−Fe bond, contributing to the absorption in the visible range (Figure S13).

**Figure 3:**
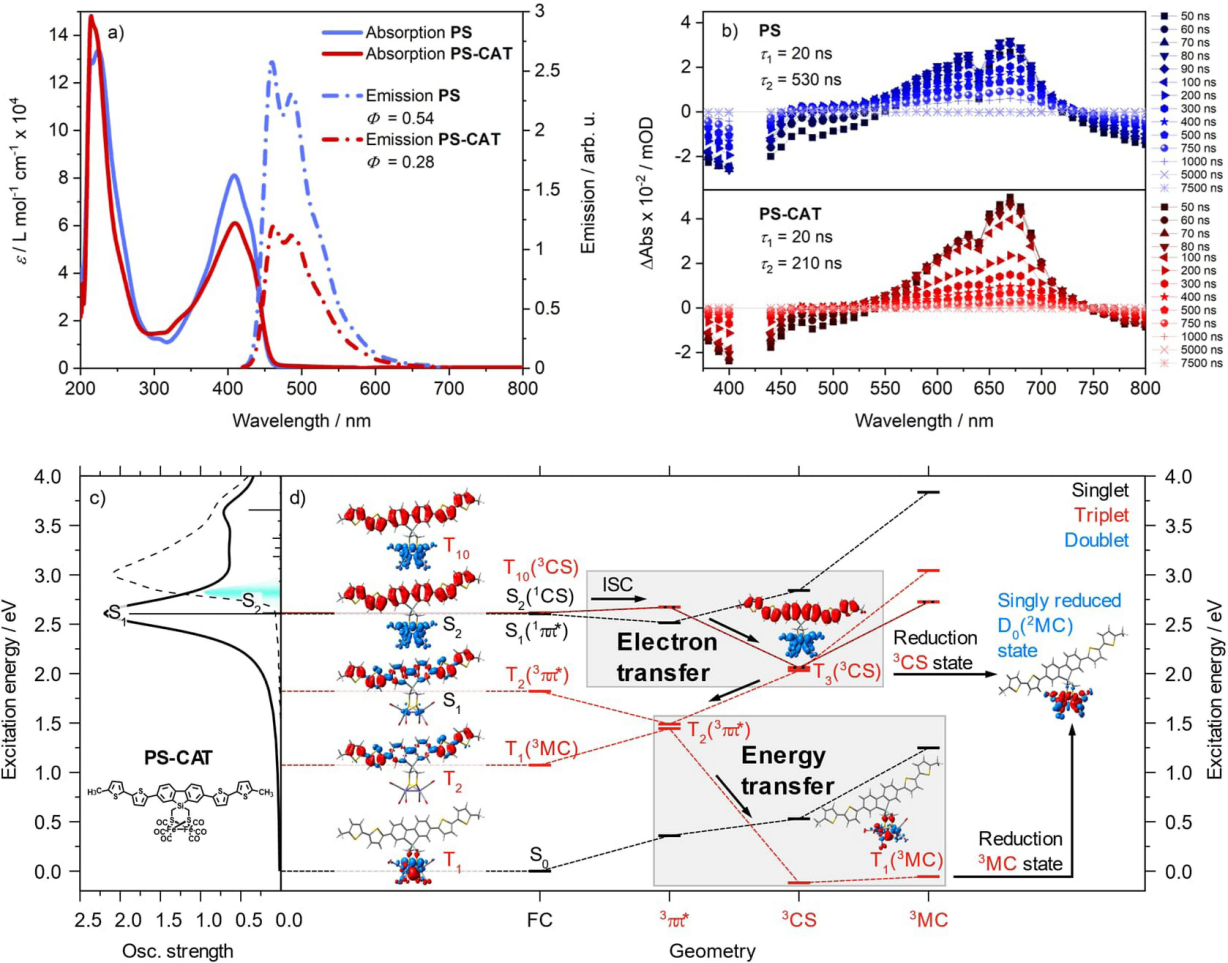
a) Steady‐state UV/Vis absorption (solid lines) of **PS** (red) and **PS‐CAT** (blue) and emission (dashed lines) in deaerated THF excited at 410 nm. b) Transient absorption spectra at indicated delay times of **PS** (top, blue) and **PS‐CAT** (bottom, red) upon excitation at 420 nm in deaerated THF. c) Experimental (black, dashed) and simulated UV/Vis absorption spectrum of **PS‐CAT** in THF; electronic transitions of interest are labelled. d) Potential excited state relaxation cascades associated to electron and energy transfer competing with radiative recombination; relative energies of involved singlet (black) and triplet states (red) are given within their respective equilibrium structures. Electron transfer and energy transfer pathways both leading to singly reduced **PS‐CAT** (D_0_, ^2^MC, blue) upon BIH reduction are highlighted. Electronic characters are indicated by charge‐density differences (CDDs, electronic transitions) and spin densities (opened‐shell ground state); charge transfer takes place from red to blue (Table S7).

Emission spectra (*λ*_exc_=410 nm) of both **PS** and **PS‐CAT** display three nearly identical vibronic peaks at 455, 484 and 515 nm (shoulder) spaced by 1250 cm^−1^, corresponding to a ground state vibrational mode coupled to the electronic transition (Table S6). The emission lifetimes were determined to 1 ns and the quantum yields are *Φ*_F_=0.54 in **PS** and *Φ*_F_=0.28 in **PS‐CAT**. The reduced emission quantum yield in **PS‐CAT** is associated with the population of a charge‐separated state (S_2_) by quantum chemical simulations. S_2_, which is not available in **PS**, is in close energetic proximity to the ππ* S_1_ state.

Nanosecond‐transient absorption spectroscopy was performed to detect the impact of the [FeFe] hydrogenase mimic on the relaxation of the triplet state populated via intersystem crossing (ISC). The data (Figure 3b, Figure S7) for **PS** and **PS‐CAT** exhibit similar spectral features. Both **PS** and **PS‐CAT** show a negative signal between 350 and 550 nm, which arises from ground‐state bleach and emission contributions between 460 and 550 nm.^[15]^ Excited state absorption (ESA) is detected at wavelengths longer than 550 nm for both **PS** and **PS‐CAT** which is characteristic for the ^3^ππ* state of the sensitizer unit.^[16]^ TDDFT simulations on the dipole‐allowed triplet‐triplet excitations of **PS‐CAT** within the equilibrium structure of the ^3^ππ* ground state, see T_2_(^3^ππ*) in Figure 3d, associate these ESA features to ^3^ππ* transitions of the excited oligothiophene at 600 and 529 nm, respectively (see T_11_ and T_16_ in Figure S15), confirming this assignment.^[17]^ At wavelengths above ≈750 nm there is a second negative signal which can be assigned to the triplet phosphorescence of the thiophenes.^[17]^ Both transient signals of **PS** and **PS‐CAT** decay biexponentially with lifetimes of *τ*_1_=20 ns (reflecting the time‐resolution of the experimental setup) and *τ*_2_=530 ns for **PS** and *τ*_1_=20 ns and *τ*_2_=210 ns for **PS‐CAT**. The fast component indicates the decay of the residual emission, the long‐lived component is associated with the decay of the ^3^ππ* state of the sensitizer unit. The shortening in *τ*_2_ reflects changes in the ^3^ππ* state lifetime of the excited sensitizer in **PS‐CAT** indicating the presence of additional relaxation channels occurring from the ^3^ππ* state with an estimated time constant of 348 ns. Nevertheless, besides the changes in lifetime of the triplet state we do not observe any absorption features at 400, 580 and 700 nm, which could be ascribed to the reduced [FeFe] moiety according to literature and UV/Vis SEC data (Figure S11/S12). Hence, no direct indication for a charge‐separated state (^3^CS, Figure 3d) is observed.[^15a^, ^18^] The absence of these features indicates either that no charge transfer takes place to reduce the Fe−Fe unit or that the CS state is too short‐lived, recombines quickly and escapes experimental detection in the absence of an electron donor.

Quantum chemical simulations indicate two relaxation pathways for **PS‐CAT**: i) electron transfer causing charge separation which is accessible from the initially excited singlet states. ii) Energy transfer deactivating the ^3^ππ* state (Figure 3d). The electron transfer pathway leads from the initially excited ^1^ππ* state via ISC to the adjacent charge‐separated T_10/3_(FC/^3^CS equilibria) state (Figure 3d), resulting in a pronounced elongation of the Fe−Fe bond (Table S4) of the formally [Fe^I^Fe^0^] active site. Upon population of the ^3^ππ* state, either via ^1^ππ*→^3^ππ* ISC or via relaxation from the ^3^CS state, an energy transfer pathway is available. In this case, hole transfer from the photooxidized photosensitizer to the photoreduced iron cluster yields an excited metal centered (^3^MC) state. During this process, the ESA decays as the initial configuration of the oligothiophene‐based chromophore is reformed (Figure S15) in agreement with the experimental observations. In this ^3^MC state cleavage of the Fe−Fe bond (Table S4) occurs as both the σ_Fe−Fe_ and the σ_Fe−Fe_* orbitals are singly populated (bond order: 0). The population of the ^3^MC state can be an explanation for the missing signatures of a CS state in the transient absorption experiment. These findings indicate that the presence of sacrificial agent is crucial for the photoinduced reduction of the [FeFe] unit, a key step of the light‐induced catalytic process. In this case, according to TDDFT simulations, both relaxation channels, i.e. electron transfer and energy transfer, yield the same singly reduced doublet species of [Fe^I^Fe^0^] character (see D_0_ spin density in Figure 3d, blue)—either upon reduction of the charge‐separated species (^3^CS) or the ^3^MC state by BIH. Alternatively, the sacrificial agent could also interfere with the described relaxation processes already in an initial stage of the relaxation cascade reducing the sensitizer directly after excitation. The quenching behaviour of the fluorescence of **PS‐CAT** by BIH gives the first indication for such a fast process (Figure S5).

The role of the sacrificial agent was elucidated by *operando* UV/Vis spectroscopy in the presence of sacrificial donor BIH (1000 equivalents) upon irradiation at 455 nm. After 30 minutes of irradiation, the absorption band of **PS‐CAT** at 403 nm in CH_3_CN/NMP (5 : 1) disappeared in favour of a characteristic band at 399 nm, which continues to build up within 17 hours (Figure S3a). Upon irradiation in the absence of the donor the absorption at 403 nm decays only slowly, indicating the light‐mediated interaction of **PS‐CAT** with BIH (Figure S3c). The newly formed species decomposed slowly in the dark and quite rapidly in the presence of oxygen (Figure S3b). It is tentatively attributed to a sensitive follow‐up product of the catalytic species, not to the catalytic species itself.

*Operando* electron paramagnetic resonance (EPR) spectroscopy reveals the formation of a reduced [FeFe] moiety under photocatalytic conditions. The catalytic mixture (30 μM **PS‐CAT**, 1000 equivalents BIH) was illuminated in the resonator at 270 K and measured at 4 K. The data show the formation of a paramagnetic [Fe^I^Fe^0^] intermediate (Figure 4), which has been postulated as key species in the light‐induced HER mechanism.^[6c]^ No signal was detected in the dark or at room temperature (Figure S8a). The *g* values (*g*_1_=2.003, *g*_2_=1.99557, *g*_3_=1.86197) derived from the EPR data are similar to those reported on [Fe^I^Fe^0^] species with the third *g* tensor being shifted further low‐field.^[19]^ This shift could be due to a low concentration of the [Fe^I^Fe^0^] intermediate in photochemical reduction, consequently leading to a lower EPR spectral resolution compared to that resulting from (electro)chemical reduction processes.[^19^, ^20^]

**Figure 4:**
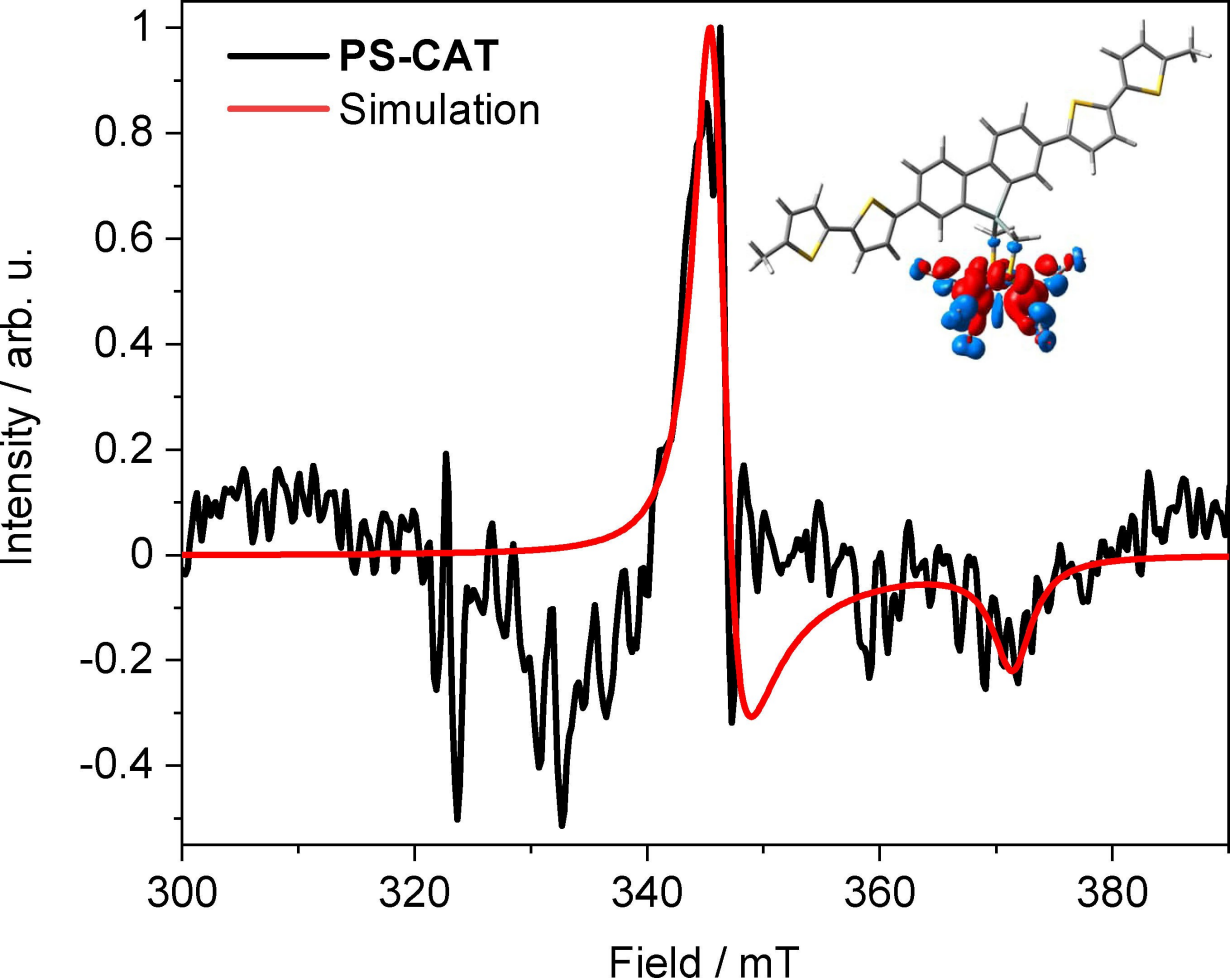
EPR spectrum of **PS‐CAT** after four hours of illumination under catalytic conditions (30 μM **PS‐CAT**). Illumination was carried out in a MD5 resonator and the EPR measurement was done at 4 K. The negative signal at 330 mT stems from resonator background. Inset: Spin density localized at the [FeFe] moiety of the singly reduced **PS‐CAT** (doublet).

In summary, we synthesized a precious metal‐free photocatalyst dyad **PS‐CAT** for photocatalytic hydrogen generation under visible light. The dyad as prototype comprising π‐conjugated oligothiophenes as light absorbers shows a remarkable, long‐term photocatalytic activity, the best reported for comparable complexes in the visible spectral range. In accordance with theory, *operando* EPR spectroscopy using BIH as sacrificial donor reveals the generation of an active [Fe^I^Fe^0^] species, which presumably drives the light‐induced hydrogen generation.

## Conflict of interest

The authors declare no conflict of interest.

## Supporting information

As a service to our authors and readers, this journal provides supporting information supplied by the authors. Such materials are peer reviewed and may be re‐organized for online delivery, but are not copy‐edited or typeset. Technical support issues arising from supporting information (other than missing files) should be addressed to the authors.

Supporting Information

Supporting Information

Supporting Information

## Data Availability

The data that support the findings of this study are available from the corresponding author upon reasonable request.
