# Supplementary material for: Activating a [FeFe] Hydrogenase Mimic for Hydrogen Evolution under Visible Light
Source: Angew Chem Int Ed Engl. 2022 Mar 23;61(20):e202202079. doi: 10.1002/anie.202202079 (PMC9313588; doi:10.1002/anie.202202079)

checkCIF/PLATON report

Structure factors have been supplied for datablock(s) masa1389

THIS REPORT IS FOR GUIDANCE ONLY. IF USED AS PART OF A REVIEW PROCEDURE FOR PUBLICATION, IT SHOULD NOT REPLACE THE EXPERTISE OF AN EXPERIENCED CRYSTALLOGRAPHIC REFEREE.

No syntax errors found. CIF dictionary Interpreting this report

Datablock: masa1389

Bond precision: C-C = 0.0050 A Wavelength=0.71073

Cell:	a=7.4430(12)	b=16.584(3)	c=19.525(3)
	alpha=102.765(2)	beta=99.356(2)	gamma=95.671(2)
Temperature:	100 K		

	Calculated	Reported
Volume	2296.4(7)	2296.4(6)
Space group	P -1	P -1
Hall group	-P 1	-P 1
Moiety formula	C48 H44 Fe2 O6 S6 Si	?
Sum formula	C48 H44 Fe2 O6 S6 Si	C48 H44 Fe2 O6 S6 Si
Mr	1048.98	1048.98
Dx,g cm-3	1.517	1.517
Z	2	2
Mu (mm-1)	0.980	0.980
F000	1084.0	1084.0
F000'	1087.37	
h,k,lmax	8,18,21	8,18,21
Nref	6432	6208
Tmin,Tmax	0.977,0.990	0.977,0.990
Tmin'	0.943	

Correction method= # Reported T Limits: Tmin=0.977 Tmax=0.990
AbsCorr = MULTI-SCAN

Data completeness= 0.965 Theta(max)= 23.055

R(reflections)= 0.0321(4907) wR2(reflections)= 0.0884(6208)

S = 1.036 Npar= 570

The following ALERTS were generated. Each ALERT has the format
test-name_ALERT_alert-type_alert-level.
Click on the hyperlinks for more details of the test.

Alert level B

```
THETM01_ALERT_3_B  The value of sine(theta_max)/wavelength is less than 0.575
            Calculated sin(theta_max)/wavelength =    0.5510
PLAT919_ALERT_3_B Reflection # Likely Affected by the Beamstop ...          1 Check
PLAT934_ALERT_3_B Number of (Iobs-Icalc)/Sigma(W) > 10 Outliers ..          3 Check
```

Alert level C

```
ABSTY02_ALERT_1_C  An _exptl_absorpt_correction_type has been given without
            a literature citation. This should be contained in the
            _exptl_absorpt_process_details field.
            Absorption correction given as multi-scan
PLAT029_ALERT_3_C _diffrn_measured_fraction_theta_full value Low .      0.965 Why?
PLAT911_ALERT_3_C Missing FCF Refl Between Thmin & STh/L=    0.551        224 Report
```

Alert level G

```
PLAT066_ALERT_1_G Predicted and Reported Tmin&Tmax Range Identical          ? Check
PLAT154_ALERT_1_G The s.u.'s on the Cell Angles are Equal ..(Note)      0.002 Degree
PLAT232_ALERT_2_G Hirshfeld Test Diff (M-X)  Fe1      --C1       .        5.5 s.u.
PLAT232_ALERT_2_G Hirshfeld Test Diff (M-X)  Fe2      --C5       .        6.0 s.u.
PLAT232_ALERT_2_G Hirshfeld Test Diff (M-X)  Fe2      --C6       .        5.5 s.u.
PLAT883_ALERT_1_G No Info/Value for _atom_sites_solution_primary .     Please Do !
PLAT909_ALERT_3_G Percentage of I>2sig(I) Data at Theta(Max) Still        68% Note
PLAT941_ALERT_3_G Average HKL Measurement Multiplicity ..........         2.8 Low
PLAT960_ALERT_3_G Number of Intensities with I  <   - 2*sig(I) ...          8 Check
PLAT965_ALERT_2_G The SHELXL WEIGHT Optimisation has not Converged     Please Check
PLAT978_ALERT_2_G Number C-C Bonds with Positive Residual Density.          4 Info
```

0 **ALERT level A** = Most likely a serious problem - resolve or explain
3 **ALERT level B** = A potentially serious problem, consider carefully
3 **ALERT level C** = Check. Ensure it is not caused by an omission or oversight
11 **ALERT level G** = General information/check it is not something unexpected

4 ALERT type 1 CIF construction/syntax error, inconsistent or missing data
5 ALERT type 2 Indicator that the structure model may be wrong or deficient
8 ALERT type 3 Indicator that the structure quality may be low
0 ALERT type 4 Improvement, methodology, query or suggestion
0 ALERT type 5 Informative message, check

It is advisable to attempt to resolve as many as possible of the alerts in all categories. Often the minor alerts point to easily fixed oversights, errors and omissions in your CIF or refinement strategy, so attention to these fine details can be worthwhile. In order to resolve some of the more serious problems it may be necessary to carry out additional measurements or structure refinements. However, the purpose of your study may justify the reported deviations and the more serious of these should normally be commented upon in the discussion or experimental section of a paper or in the "special_details" fields of the CIF. checkCIF was carefully designed to identify outliers and unusual parameters, but every test has its limitations and alerts that are not important in a particular case may appear. Conversely, the absence of alerts does not guarantee there are no aspects of the results needing attention. It is up to the individual to critically assess their own results and, if necessary, seek expert advice.

Publication of your CIF in IUCr journals

A basic structural check has been run on your CIF. These basic checks will be run on all CIFs submitted for publication in IUCr journals (*Acta Crystallographica*, *Journal of Applied Crystallography*, *Journal of Synchrotron Radiation*); however, if you intend to submit to *Acta Crystallographica Section C* or *E* or *IUCrData*, you should make sure that full publication checks are run on the final version of your CIF prior to submission.

Publication of your CIF in other journals

Please refer to the *Notes for Authors* of the relevant journal for any special instructions relating to CIF submission.

PLATON version of 13/07/2021; check.def file version of 13/07/2021

Datablock masa1389 - ellipsoid plot

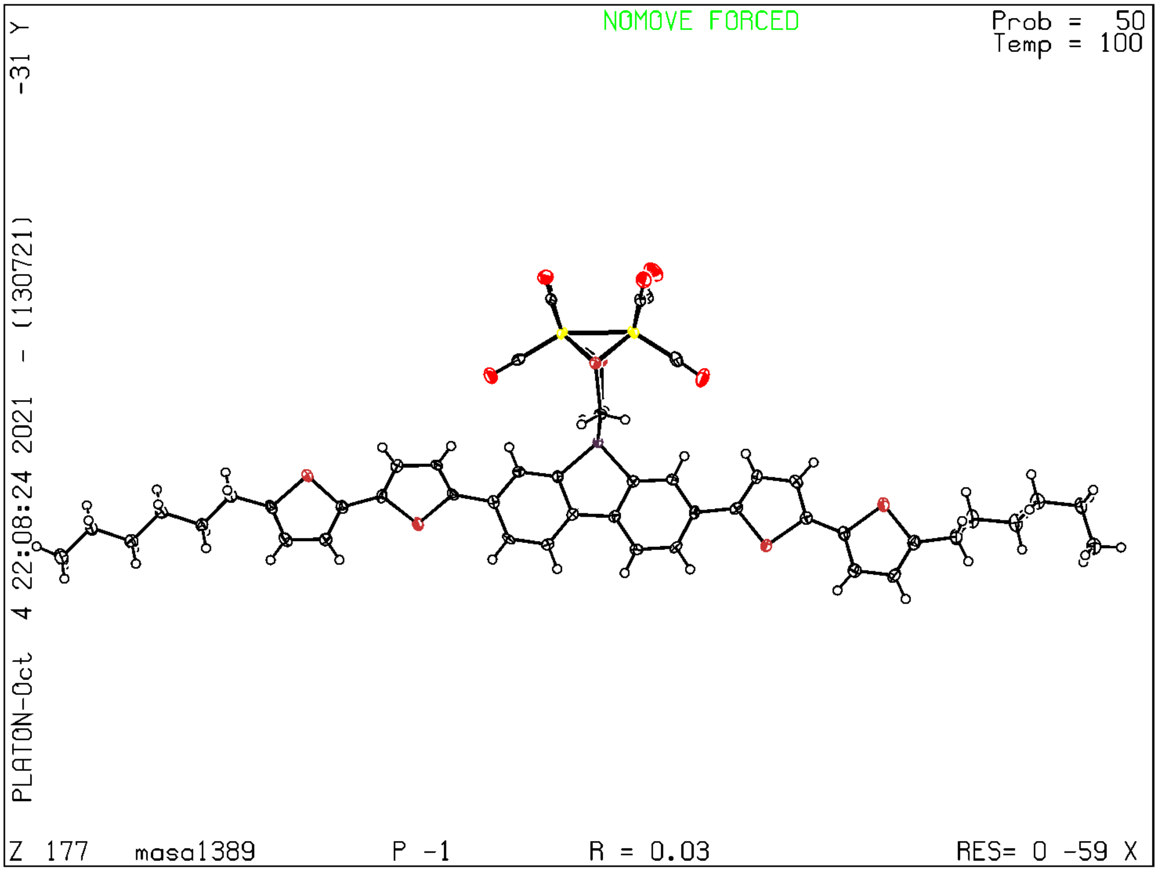

Supplement: Supplementary file 1 — Supporting Information [file ANIE-61-0-s003.pdf]
