# Supplementary material for: Activating a [FeFe] Hydrogenase Mimic for Hydrogen Evolution under Visible Light
Source: Angew Chem Int Ed Engl. 2022 Mar 23;61(20):e202202079. doi: 10.1002/anie.202202079 (PMC9313588; doi:10.1002/anie.202202079)
Supplement: Supplementary file 3 — Supporting Information [file ANIE-61-0-s002.pdf]

Angewandte Chemie
Eine Zeitschrift der Gesellschaft Deutscher Chemiker

Supporting Information

Activating a [FeFe] Hydrogenase Mimic for Hydrogen Evolution under Visible Light

P. Buday, C. Kasahara, E. Hofmeister, D. Kowalczyk, M. K. Farh, S. Riediger, M. Schulz, M. Wächtler, S. Furukawa, M. Saito, D. Ziegenbalg, S. Gräfe, P. Bäuerle, S. Kupfer*, B. Dietzek-Ivanšić*, W. Weigand**

Table of Contents

Materials and instrumentation 3
Synthesis and characterization 6
NMR spectra 11
Single crystal X-ray structure determination 16
Photocatalysis 19
UV-vis-monitored photocatalysis 21
Fluorescence quenching in presence of BIH 22
Time-resolved spectroscopy 25
EPR spectroscopy 27
Electrochemical characterization 28
Computational details 32
References 39
Author Contributions 40

Materials and instrumentation

The following materials were synthesized according to the corresponding literature procedures: 2,2'-dibromo-[1,1'-biphenyl]-4,4'-diamine (**1**),[1] 2-(5'-Hexyl-2,2'-bithien-5-yl)-4,4,5,5-tetramethyl-1,3,2-dioxaborolan,[2] $SiCl_2(CH_2Cl)_2$,[3] 1,3-Dimethyl-2-phenylbenzimidazoline (BIH) from 2-phenyl-1H-benzo[d]imidazole.[4] All other chemicals and solvents were commercially available (Sigma-Aldrich, Acros, TCI, Alfa Aesar, abcr). Column chromatography was performed with Geduran Si 60 (0.063-0.200 mm), TLC monitoring on TLC Silica gel 60 F254.

Melting points: Melting points were determined with an APOTEC apparatus and are uncorrected.

NMR: NMR spectra were recorded with Bruker 600 MHz and 400 MHz spectrometers (Avance III).

IR: IR spectroscopy was performed with a Tensor 27 FT-IR spectrometer.

MS: High resolution mass spectrometry was carried out using a Thermo Scientific Q Exactive plus Orbitrap mass spectrometer coupled to an atmospheric pressure chemical ionization source (APCI). EI spectra were measured on a Finnigan MAT SSQ710 using the direct evaporation probe (DEP) technique and electron ionization (EI) at 70 eV.

Elemental analysis: For Elemental analysis an Euro Vector EA3000 element Analyser was utilized.

X-ray: The intensity data were collected on a Bruker Apex II diffractometer using graphite-monochromated Mo-K_a radiation. Data were corrected for Lorentz and polarization effects; absorption was taken into account on a semi-empirical basis using multiple scans. Structure refinement made use of SHELXL.[5] Hydrogen atoms were included at calculated positions with isotropic displacement parameters.

Photocatalysis / UV-vis: Photocatalytic investigations with subsequent hydrogen quantification (7820A GC-TCD; Agilent Technologies) took place in a novel standard, 3D printed photoreactor utilizing a M455L3 Mounted LED from Thorlabs connected with a COP1-A Collimation Adapter (Thorlabs) (LED power: ≈ 10 mW cm^{-2}). For monitoring photocatalysis by UV-vis spectroscopy, a V-780 Spectrophotometer (JASCO) was used to record the spectra between irradiation intervals with a M455L3 Mounted LED connected with a collimation lens (effective LED power: ≈ 180 mW).

Utilized Photoreactor: Photocatalytic experiments were carried out in a custom made, modular 3D printed, photoreactor platform. The irradiation chamber of the reactor had the dimensions of 90x90x100 mm (Figure S1 (i)). The photoreactor was manufactured form polylactic acid using Fused Deposition Modeling. Due to its reflecting properties in the visible wavelength range, white filament was used. The irradiation chamber consisted of two walls with slots (Figure S1 (ii)), in which a sample holder can be installed. Circularly arranged vials can be irradiated from the bottom with an irradiation module at a defined (adjustable) distance from the light source. 8 4 mL GC-vials were placed in a circular holder with a diameter of 46 mm (measured from the center of two opposing vials). The holder was placed in the third slot (distance 30 mm) measured from the bottom of the irradiation module. At the bottom an adapter module for a Thorlabs collimator was installed together with a 455 nm LED (M455L3 Mounted LED from Thorlabs) (Figure S1 (iii)). The LED was operated at (100 %) of its maximum power. A third wall of the corpus was equipped with an opening. Here, a fan was installed to temper the inner of the irradiation chamber (Figure S1 (iv)). A fourth wall is used to close the reactor. It is equipped with an opening which works as exhaust for the ventilation air (Figure S1 (v)). The reaction chamber was ventilated using a fan (26.43 L s^{-1}) to avoid heat accumulation in the reaction chamber. The reactor was closed from above using a cover (Figure S1 (vi)). The complete modular reactor platform is depicted in Figure S1 (vii).

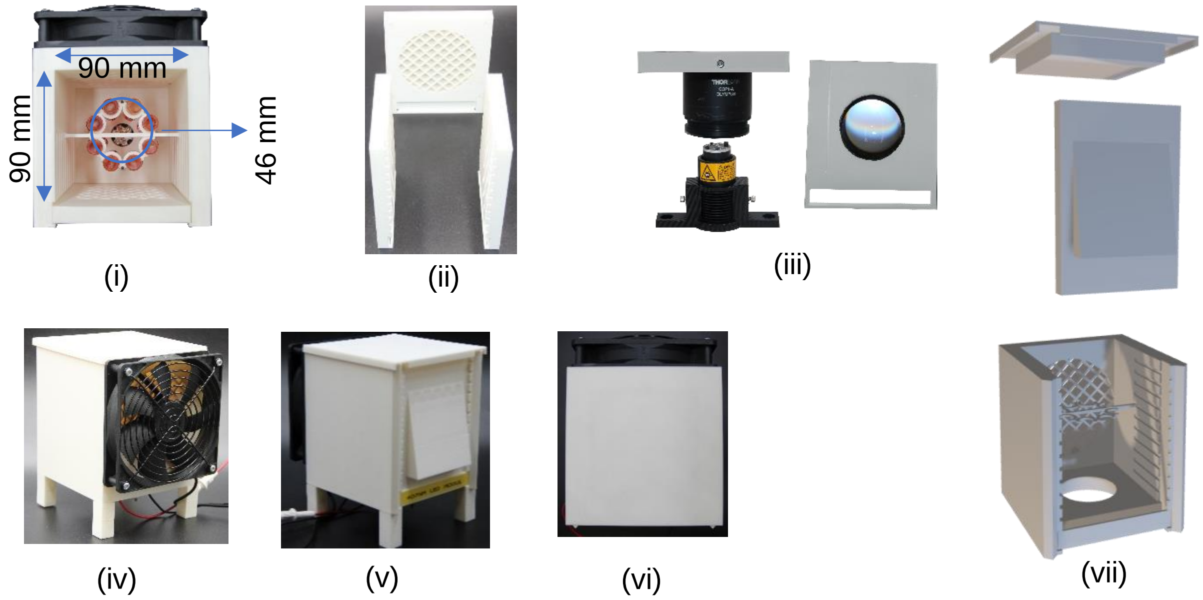

Figure S1: Modular photoreactor platform: irradiation chamber (i), side walls (ii), adapter module for Thorlabs collimator (iii), back wall with fan (iv), front wall with ventilation opening (v), reactor cover (vi) and model of whole reactor setup (vii).

Steady-state spectroscopy: UV-vis absorption spectroscopy was carried out with a V-780 Spectrophotometer (JASCO) and a SPECORD S600 (Analytik Jena). Emission spectroscopy and emission quenching experiments were performed with a FLS980 Spectrometer (Edinburgh Instruments), absolute fluorescence quantum yields were determined with an FLS980 Integrating Sphere (Edinburgh Instruments).

Time-resolved spectroscopy:

Streak camera – The two-dimensional (time and wavelength) emission decay profiles were measured using a Hamamatsu streak scope C4334 in photon counting mode using a time window of 500 ns. After excitation with the frequency-doubled output of a Ti-sapphire laser (Tsunami, Newport Spectra-Physics GmbH; pulse-to-pulse repetition rate 400 kHz after passing a pulse selector, model 3980, Newport Spectra-Physics GmbH) at λ_{ex} = 460 nm, the luminescence of the sample was collected in a 90°-arrangement.

Nanosecond transient absorption – The excitation light source for measurements on the submicro-second time scale is based on a Nd:YAG laser (Continuum Surelite), yielding 5 ns pulses at 1064 nm with a repetition rate of 10 Hz. The fundamental pump pulses were frequency-tripled (yielding 355 nm), which was used as input for generating pump pulses centered at 420 nm using an optical parametric oscillator (Continuum Surelite). The kinetics were studied using a probe light provided by a 75 W xenon arc lamp, which was focused on the sample by a concave mirror. After passing through the sample, the probe pulses were spectrally dispersed (Acton Princeton Instrument 2300), detected on a photomultiplier (Hamamatsu R928), and processed (Pascher Instruments AB). TA kinetics were detected as a single-wavelength kinetic between 380 and 800 nm in steps of 10 nm. For all measurements, the power of the pump beam was kept at 0.35 mJ. A long pass filter (435 nm) to eliminate the pump scattering was used at wavelengths above 430 nm.

EPR: A X-Band ELEXSYS E500 spectrometer from Bruker with HE-flow cryostat equipped with a MD5 resonator at 5 K was used for EPR measurements. Illumination was carried out at room temperature at 420 nm. The samples were frozen under illumination. All experiments were carried out in an EPR tube for low temperature measurements (3 mm diameter). Simulation of experimental data was carried out with EasySpin in MatLab.[6]

Electrochemistry:

Cyclic voltammetry (CV) – Basic CV was accomplished with a three-electrode setup using a glassy carbon (*d* = 1.6 mm) working electrode, a Ag^+/Ag (in acetonitrile) reference electrode and a Pt (wire) counter electrode, driven by a Reference 600 Potentiostat/Galvanostat/ZRA from Gamry Instruments.

Spectroelectrochemistry (SEC) – The measurements were executed with a BioLogic SP-50 Potentiostat. IR SEC was carried out in a Specac Omni-Cell with a Pt (mesh) working and counter electrode and an Ag (wire) reference electrode using a Tensor 27 FT-IR spectrometer. UV-vis SEC was done with a glassy carbon working electrode, a Pt (wire) counter electrode and an Ag (wire) reference electrode making use of a SPECORD S600 UV-vis spectrometer. The glassy carbon electrode has a hole cut in to ensure maximum concentration of the desired compound during measurements.

Synthesis and characterization

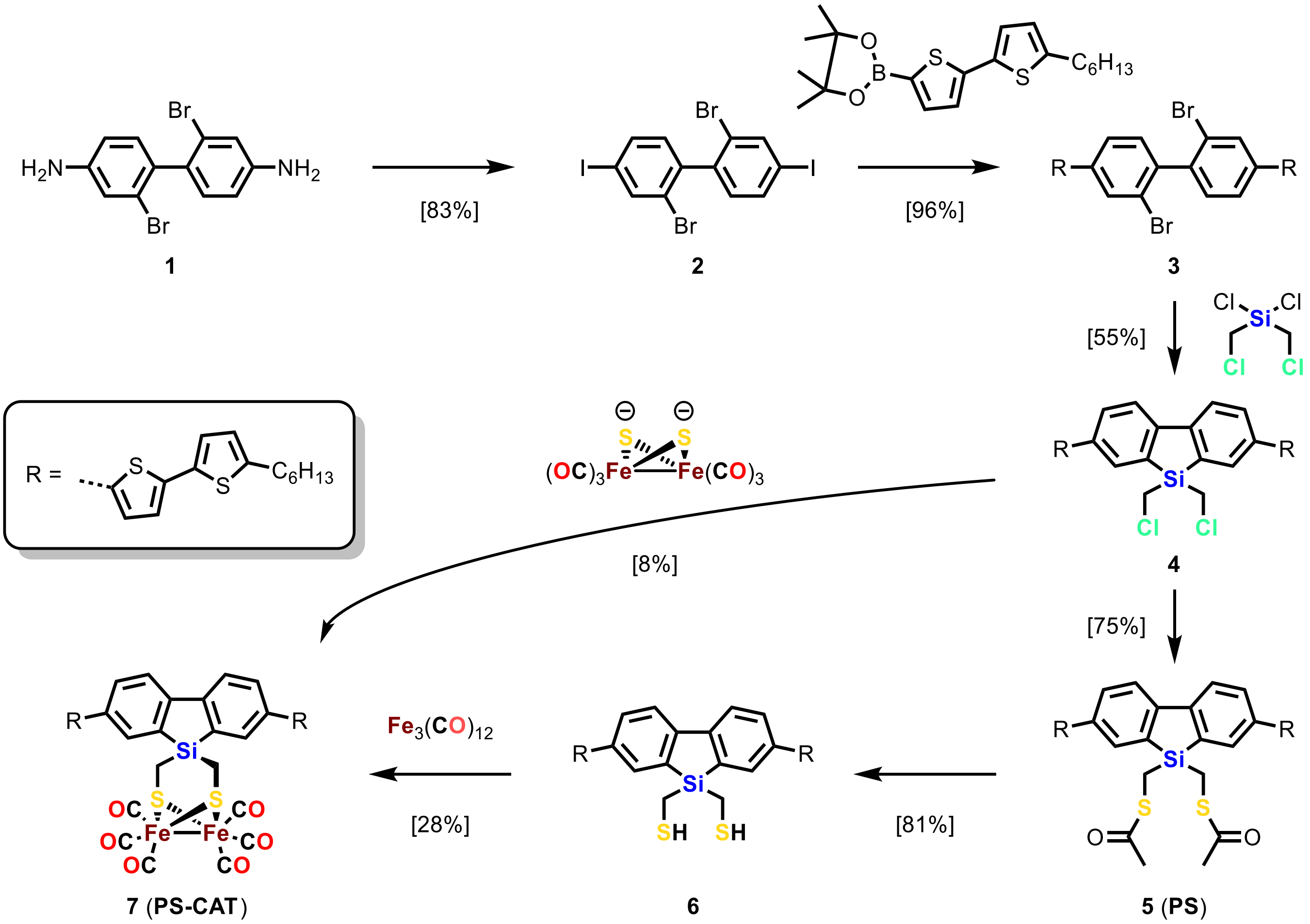

Scheme S1: Synthetic pathway to **7** (**PS-CAT**) *via* **5** (**PS**).

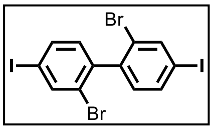

2,2'-dibromo-4,4'-diiodo-1,1'-biphenyl (**2**). Referring to literature.[1, 7] 2,2'-dibromo-[1,1'-biphenyl]-4,4'-diamine (**1**) (0.95 g, 2.8 mmol) was dissolved in diethyl ether (20 mL) and hydrochloric acid (37 %, 40 mL) was added dropwise. After adding water (30 mL), ice (50 g) and hydrochloric acid (37 %, 10 mL), the reaction mixture was cooled to 0 °C. A solution of sodium nitrite (1 g, 14.5 mmol) in water (5 mL) was added dropwise and the resulting reddish solution was stirred at 0 °C for 5 minutes, before a solution of potassium iodide (4 g, 24.1 mmol) in water (10 mL) was added within 1 minute. The brown suspension was stirred at 0 °C for 1 hour, next at room temperature for 30 minutes and then refluxed for 1.5 hours. Subsequently, the mixture was allowed to cool down to room temperature and sodium thiosulfate was added to afford a pale-yellow solution. The aqueous phase was extracted with dichloromethane (2x 50 mL) and the organic phase was dried over Na_2SO_4. The solvent was removed under reduced pressure and **2** (1.30 g, 2.3 mmol, 83 %) was obtained as colourless solid after wet silica column chromatography using petroleum ether as eluent.

^{1}H NMR (400 MHz, DMSO-d$_6$): δ = 7.09 (d, *J*=7.89 Hz, 2 H), 7.83 (dd, *J*=7.89, 1.75 Hz, 2 H), 8.12 ppm (d, *J*=1.46 Hz, 2 H).

^{13}C{^{1}H} NMR (101 MHz, DMSO-d$_6$): δ = 95.5, 123.7, 132.5, 136.6, 139.7, 140.2 ppm.

MS (EI, 70 eV): *m*/*z* (%) = 564 (100) [*M*]$^+$.

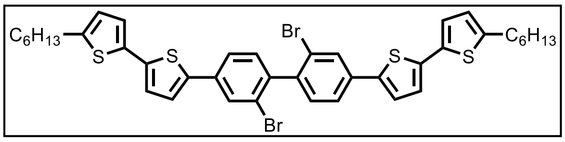

5',5'''-(2,2'-dibromo-[1,1'-biphenyl]-4,4'-diyl)bis(5-hexyl-2,2'-bithiophene) (**3**). The reaction was performed under an argon atmosphere. In a three-necked flask 2,2'-dibromo-4,4'-diiodo-1,1'-biphenyl (**2**) (1.33 g, 2.37 mmol), 2-(5'-hexyl-[2,2'-bithiophen]-5-yl)-4,4,5,5-tetramethyl-1,3,2-dioxaborolane (2.12 g, 5.64 mmol, 2.38 eq), benzyltriethylammonium chloride (1.3 g, 5.7 mmol, 2.42 eq) and [$Pd(PPh_3)_4$] (816 mg, 0.71 mmol, 15 mol%) were dissolved in dry toluene (40 mL). After heating to 60 °C, K_2CO_3 (23 mL of 2 M solution, 46 mmol) was added and the solution was refluxed for 12 hours. The heating bath was removed and the reaction mixture allowed to cool down to room temperature, followed by the addition of water (20 mL). Subsequently, dichloromethane (80 mL) was added and the organic layer was separated. The latter was washed with brine and water and then dried over Na_2SO_4. After removing the solvent under reduced pressure, the residue was purified by wet silica gel filtration using *n*-hexane for removing any impurities and then a mixture of *n*-haxane/dichloromethane = 3:1 to get **3** (1.84 g, 2.28 mmol, 96 %) as light yellow solid.

R_f = 0.76 (*n*-hexane/CH_2Cl_2 = 2:1).

m. p.: 135-136 °C.

^1H NMR (400 MHz, CD_2Cl_2): δ = 0.91 (t, *J*=7.02 Hz, 6 H (overlap with *n*-hexane), hexyl), 1.29 – 1.46 (m, 12 H (overlap with *n*-hexane), hexyl), 1.70 (quin, *J*=7.45 Hz, 4 H, hexyl), 2.82 (t, *J*=7.60 Hz, 4 H, hexyl), 6.73 (d, *J*=3.80 Hz, 2 H, het-Ar), 7.06 (d, *J*=3.51 Hz, 2 H, het-Ar), 7.11 (d, *J*=3.80 Hz, 2 H, het-Ar), 7.28 (d, *J*=7.89 Hz, 2 H, Ar), 7.31 (d, *J*=3.80 Hz, 2 H, het-Ar), 7.61 (dd, *J*=8.04, 1.90 Hz, 2 H, Ar), 7.93 ppm (d, *J*=1.75 Hz, 2 H).

$^{13}C\{^1H\}$ NMR (101 MHz, CD_2Cl_2): δ = 14.4, 23.2, 29.3, 30.7, 32.2, 32.2, 124.3, 124.5, 124.6, 124.7, 125.5, 125.6, 129.6, 132.1, 134.8, 136.2, 139.0, 140.4, 140.8, 146.8 ppm.

HRMS (APCI+): *m*/*z* calcd for $C_{40}H_{41}Br_2S_4^+$ ($[M+H]^+$): 807.0452; found: 807.0447.

Elemental analysis calcd (%) for $C_{40}H_{40}Br_2S_4$: C 59.40, H 4.99, S 15.86; found C 58.89, H 4.87, S 15.63.

IR: $\tilde{\nu}$ = 3060 (w), 2953 (w), 2922 (m), 2852 (m), 1594 (m), 1488 (m), 1444 (m), 1377 (m), 1199 (m), 1084 (m), 1040 (m), 997 (m), 970 (m), 864 (m), 779 (s), 746 (m), 667 (m) cm^{-1}.

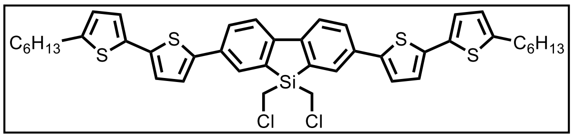

5,5-bis(chloromethyl)-3,7-bis(5'-hexyl-[2,2'-bithiophen]-5-yl)-5H-dibenzo[b,d]silole (**4**). Referring to literature.[8] The reaction was performed under nitrogen gas. *n*-BuLi (2.1 mL, 2.5 M in hexane, 5.21 mmol, 2.03 eq) was added dropwise within 2 minutes to a stirred suspension of **3** (2.07 g, 2.57 mmol) in THF (30 mL) at -78 °C. After addition the suspension cleared up and then another suspension occurred. The temperature was held at -78 °C for 30 minutes and after that at room temperature for 10 minutes. The reaction mixture was cooled again to -78 °C and stirred for 30 minutes, followed by the addition of $SiCl_2(CH_2Cl)_2$ (0.5 mL, 3.73 mmol, 1.45 eq) in THF (5 mL) within 3 minutes. The suspension was allowed to slowly warm up to room temperature overnight resulting in a dark reddish-brown solution with some precipitate. Dry silica was added and the solvent was evaporated under reduced pressure. Wet silica column chromatography (different mixtures of $CHCl_3$/*n*-hexane and toluene/*n*-hexane) did not afford pure, yellow **4** (about 1.10 g, 1.42 mmol, 55 %) since some side product could not be removed. In another unsuccessful trial an

excess of *n*-BuLi (4 eq) and $SiCl_2(CH_2Cl)_2$ (12.14 eq) was used to avoid the formation of any side product, so **4** was used without further purification.

R_f = 0.67 (*n*-hexane/$CHCl_3$ = 2:1).

^{1}H NMR (400 MHz, $CDCl_3$): δ = 0.91 (t, *J*=6.87 Hz, 6 H (overlap with *n*-hexane), hexyl), 1.29 – 1.47 (m, 12 H (overlap with *n*-hexane), hexyl), 1.70 (quin, *J*=7.45 Hz, 4 H (overlap with *n*-hexane), hexyl), 2.81 (t, *J*=7.45 Hz, 4 H (overlap with *n*-hexane), hexyl), 3.37 (s, 4 H, Si*CH*$_2$), 6.71 (d, *J*=3.51 Hz, 2 H, het-Ar), 7.04 (d, *J*=3.51 Hz, 2 H, het-Ar), 7.09 (d, *J*=3.80 Hz, 2 H, het-Ar), 7.25 – 7.29 (d (overlap with $CDCl_3$), *J*=3.80 Hz, 2 H, het-Ar), 7.71 (dd, *J*=8.18, 1.75 Hz, 2 H, het-Ar), 7.76 – 7.83 (m, 2 H, Ar), 8.00 ppm (d, *J*=1.75 Hz, 2 H, Ar).

$^{13}C\{^1H\}$ NMR (151 MHz, $CDCl_3$): δ = 14.1, 22.6, 25.2 (Si*C*H_2), 28.8, 30.2, 31.6, 121.7, 123.4, 123.9, 124.0, 124.9, 129.0, 131.2, 132.7, 133.7, 134.7, 137.7, 141.7, 145.7, 147.1 ppm.

HRMS (APCI+): *m/z* calcd for $C_{42}H_{45}Cl_2S_4Si^+$ ([M+H]$^+$): 775.1556; found: 775.1545.

IR: $\tilde{\nu}$ = 3067 (w), 2952 (w), 2922 (m), 2853 (m), 2360 (w), 1600 (w), 1486 (m), 1456 (m), 1379 (m), 1055 (m), 871 (m), 792 (s), 723 (s), 695 (m), 633 (m) cm^{-1}.

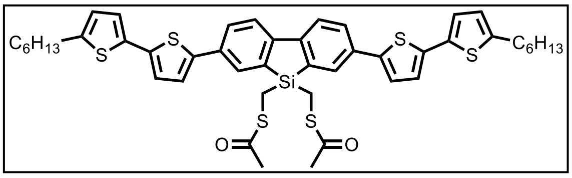

S,S'-((3,7-bis(5'-hexyl-[2,2'-bithiophen]-5-yl)-5H-dibenzo[b,d]silole-5,5-diyl)bis(methylene)) diethanethioate (**5**). Referring to literature.[9] **4** (693 mg, 0.90 mmol) was added as solid to a suspension of potassium thioacetate (340 mg, 2.98 mmol, 3.3 eq) in THF (40 mL) and the orange suspension was stirred at room temperature for 20 hours. The solvent was removed by a nitrogen flow and the residue dissolved in a mixture of *n*-hexane/toluene (4:1). Wet silica column chromatography (*n*-hexane/toluene = 4:1 to pure toluene) afforded **5** (570 mg, 0.67 mmol, 75 %) as yellow solid.

R_f = 0.54 (toluene).

m. p.: 96.5-97.5 °C.

^{1}H NMR (400 MHz, $CDCl_3$): δ = 0.92 (t, *J*=6.87 Hz, 6 H, hexyl), 1.25 – 1.47 (m, 12 H (overlap with *n*-hexane), hexyl), 1.64 – 1.77 (m, 4 H, hexyl), 2.35 (s, 6 H, CO*CH*$_3$), 2.57 (s, 4 H, Si*CH*$_2$), 2.82 (t, *J*=7.45 Hz, 4 H, hexyl), 6.71 (d, *J*=3.51 Hz, 2 H, het-Ar), 7.04 (d, *J*=3.51 Hz, 2 H, het-Ar) 7.09 (d, *J*=3.80 Hz, 2 H, het-Ar) 7.27 (d, *J*=3.51 Hz, 2 H, het-Ar) 7.65 (dd, *J*=8.18, 1.75 Hz, 2 H, Ar) 7.70 - 7.78 (m, 2 H, Ar) 7.94 ppm (d, *J*=1.75 Hz, 2 H, Ar).

$^{13}C\{^1H\}$ NMR (101 MHz, $CDCl_3$): δ = 10.2 (Si*C*H_2), 14.1, 22.6, 28.8, 30.1, 30.2, 31.6, 121.5, 123.4, 123.8, 123.9, 124.8, 128.4, 130.7, 133.4, 134.8, 135.1, 137.5, 142.1, 145.6, 147.0, 196.2 ppm (*C*OCH_3).

HRMS (APCI+): *m/z* calcd for $C_{46}H_{51}O_2S_6Si^+$ ([M+H]$^+$): 855.1977; found: 855.1970.

Elemental analysis calcd (%) for $C_{46}H_{50}O_2S_6Si$: C 64.59, H 5.89, S 22.49; found C 64.41, H 5.85, S 22.55.

IR: $\tilde{\nu}$ = 3066 (w), 2922 (m), 2853 (m), 2361 (w), 2334 (w), 1694 (m), 1674 (m), 1456 (m), 1352 (m), 1138 (m), 1056 (m), 953 (m), 825 (m), 789 (vs), 617 (s) cm^{-1}.

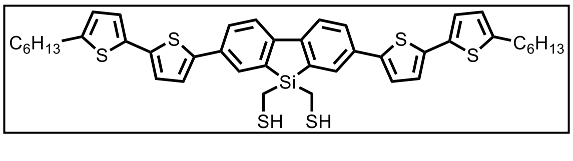

(3,7-bis(5'-hexyl-[2,2'-bithiophen]-5-yl)-5H-dibenzo[b,d]silole-5,5-diyl)dimethanethiol (**6**). Referring to literature.[9] The reaction was carried out under an Ar

atmosphere. **5** (275 mg, 0.32 mmol) was added as solid to a cooled suspension of lithium aluminium hydride (550 mg, 14.5 mmol, 45 eq) in diethyl ether (80 mL) at 0 °C. The cold bath was removed and the greenish yellow suspension was stirred at room temperature for 12 hours. After cooling to 0 °C, hydrochloric acid (2 M, 10 mL) was added carefully, followed by the addition of chloroform. The organic phase was separated and the aqueous phase extracted two times with chloroform. The combined organic solutions were dried over sodium sulfate and the solvent was removed under reduced pressure. Wet silica gel filtration ($CHCl_3$) afforded **6** (200 mg, 0.26 mmol, 81 %) as yellow solid, which was used without further purification.

^{1}H NMR (400 MHz, $CDCl_3$): *δ* = 0.91 (t, *J*=6.58 Hz, 6 H (overlap with *n*-hexane), hexyl), 1.28 – 1.43 (m, 12 H (overlap with *n*-hexane), hexyl), 1.47 (t, *J*=7.75 Hz, 2 H, S*H*), 1.70 (quin, *J*=7.38 Hz, 4 H (overlap with *n*-hexane), hexyl), 2.23 (d, *J*=7.89 Hz, 4 H, SiC*H*$_2$), 2.82 (t, *J*=7.60 Hz, 4 H (overlap with *n*-hexane), hexyl), 6.71 (d, *J*=3.51 Hz, 2 H, het-Ar), 7.04 (d, *J*=3.22 Hz, 2 H, het-Ar), 7.10 (d, *J*=3.51 Hz, 2 H, het-Ar), 7.28 (d (in overlap with $CDCl_3$), 2 H, het-Ar), 7.71 (d, *J*=8.18 Hz, 2 H, Ar), 7.81 (d, *J*=8.18 Hz, 2 H, Ar), 7.99 ppm (s, 2 H, Ar).

^{13}C{^{1}H} NMR (101 MHz, $CDCl_3$): δ = 3.0 (Si*C*H$_2$) 14.1, 22.6, 28.8, 30.2, 31.6, 121.6, 123.4, 123.9, 124.9, 128.5, 130.7, 133.5, 134.6, 134.7, 137.6, 141.9, 145.7, 147.2 ppm.

HRMS (APCI+): *m*/*z* calcd for $C_{42}H_{47}S_6Si^+$ ($[M+H]^+$): 771.1766; found: 771.1758.

IR: $\tilde{\nu}$ = 2921 (w), 2852 (w), 2361 (w), 2334 (w), 1456 (w), 1376 (w), 1261 (w), 1055 (w), 871 (w), 814 (w), 792 (m), 774 (m), 724 (m) cm^{-1}.

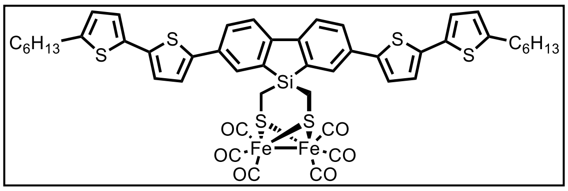

[($C_{42}H_{44}S_4SiS_2$)$Fe_2(CO)_6$] (**7**). Referring to literature.[8] **6** (58 mg, 81 µmol) was dissolved in a mixture of toluene and *N*-Methyl-2-pyrrolidone (NMP) (20:1, 21 mL). $Fe_3(CO)_{12}$ (80 mg, 159 µmol, 2 eq) was added as solid and the resulting green solution was stirred at room temperature for 12 hours. The reddish solution was concentrated to a small volume with a nitrogen flow and the residue was purified by column chromatography (*n*-hexane/toluene = 1:1). **6** (23 mg, 28 %) was obtained as orange brown solid.

In another protocol, referring to literature,[10] $LiBHEt_3$ (1 M in THF, 2.49 mmol, 2.5 eq) was added dropwise to a stirred solution of [(*µ*-S)$_2$Fe$_2$(CO)$_6$] (428 mg, 1.24 mmol, 1 eq) in THF (50 mL) at -78 °C. The temperature was held at -90 °C for 2 hours and after that at room temperature for 5 minutes. The reaction mixture was cooled again to -90 °C, followed by the addition of **4** (955 mg, 1.23 mmol,1 eq) in THF (32 mL) and subsequently was allowed to slowly warm up to room temperature overnight. After solvent removal under reduced pressure, the residue was dissolved in chloroform. The organic phase was washed with water, then dried over magnesium sulfate and removed in vacuo. The crude material was purified by flash wet silica column chromatography (*n*-hexane/$CHCl_3$ = 3:1 to 1:1) and further by gel permeation chromatography. The product was washed with *n*-hexane and obtained as orange powder (104 mg, 0.099 mmol, 8 %).

R_f = 0.58 (*n*-hexane/toluene = 2:1).

^{1}H NMR (400 MHz, $CDCl_3$): *δ* = 0.87 – 0.96 (m, 6 H (overlap with *n*-hexane), hexyl), 1.22 – 1.47 (m, 12 H (overlap with *n*-hexane), hexyl), 1.66 – 1.78 (m, 4 H (overlap with *n*-hexane), hexyl), 2.00 (s, 4 H, SiC*H*$_2$), 2.83 (t, *J*=7.60 Hz, 4 H, hexyl), 6.73 (d, *J*=3.51 Hz, 2 H, het-Ar), 7.05 (d, *J*=3.51 Hz, 2 H, het-Ar), 7.11 (d, *J*=3.80 Hz, 2 H, het-Ar), 7.25 (d, *J*=3.80 Hz, 2 H, het-Ar), 7.66 (dd, *J*=8.20, 1.80 Hz, 2 H, Ar), 7.72 (d, *J*=8.2 Hz, 2 H, Ar), 7.97 ppm (d, *J*=1.75 Hz, 2 H, Ar).

$^{13}C\{^{1}H\}$ NMR (101 MHz, $CDCl_3$): δ = 1.7 (Si*C*H_2), 14.1, 22.6, 28.8, 30.2, 31.6, 121.5, 123.5, 123.9, 124.0, 124.9, 128.4, 129.8, 134.1, 134.7, 136.1, 137.8, 141.6, 145.8, 146.1, 207.3 ppm (*C*O).

MS: Applied mass spectrometric techniques (APCI, EI) gave no molecular ion or significant fragment peaks.

Elemental analysis calcd (%) for $C_{48}H_{44}Fe_2O_6S_6Si$: C 54.96, H 4.23, S 18.34; found C 55.51, H 4.28, S 18.14.

IR: $\tilde{\nu}$ = 3065 (w), 2927 (m), 2854 (m), 2361 (w), 2070 (s), 2019 (s), 1982 (vs), 1961 (vs), 1484 (m), 1135 (m), 1057 (m), 872 (w), 789 (s), 720 (m), 620 (m), 606 (m) cm^{-1}.

NMR spectra

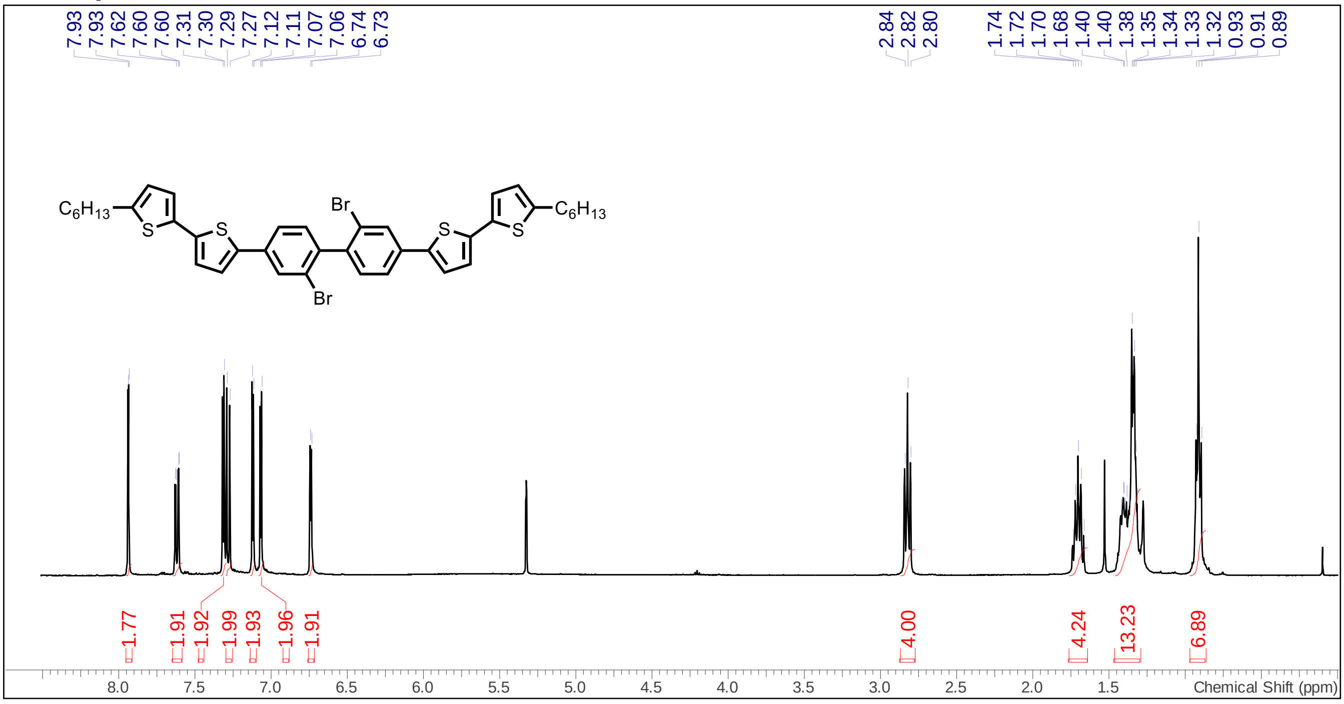

^{1}H NMR spectrum (CD_2Cl_2, 400 MHz) of **3**.

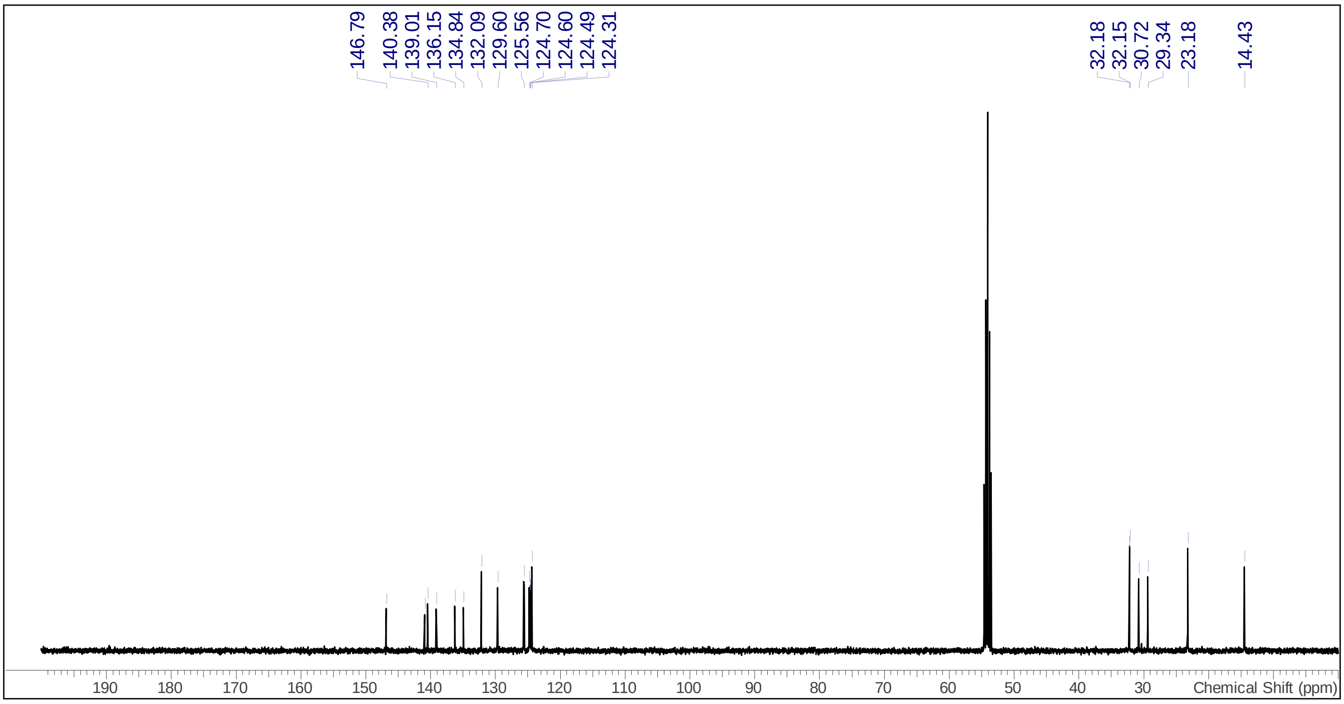

^{13}C{^{1}H} NMR spectrum (CD_2Cl_2, 101 MHz) of **3**.

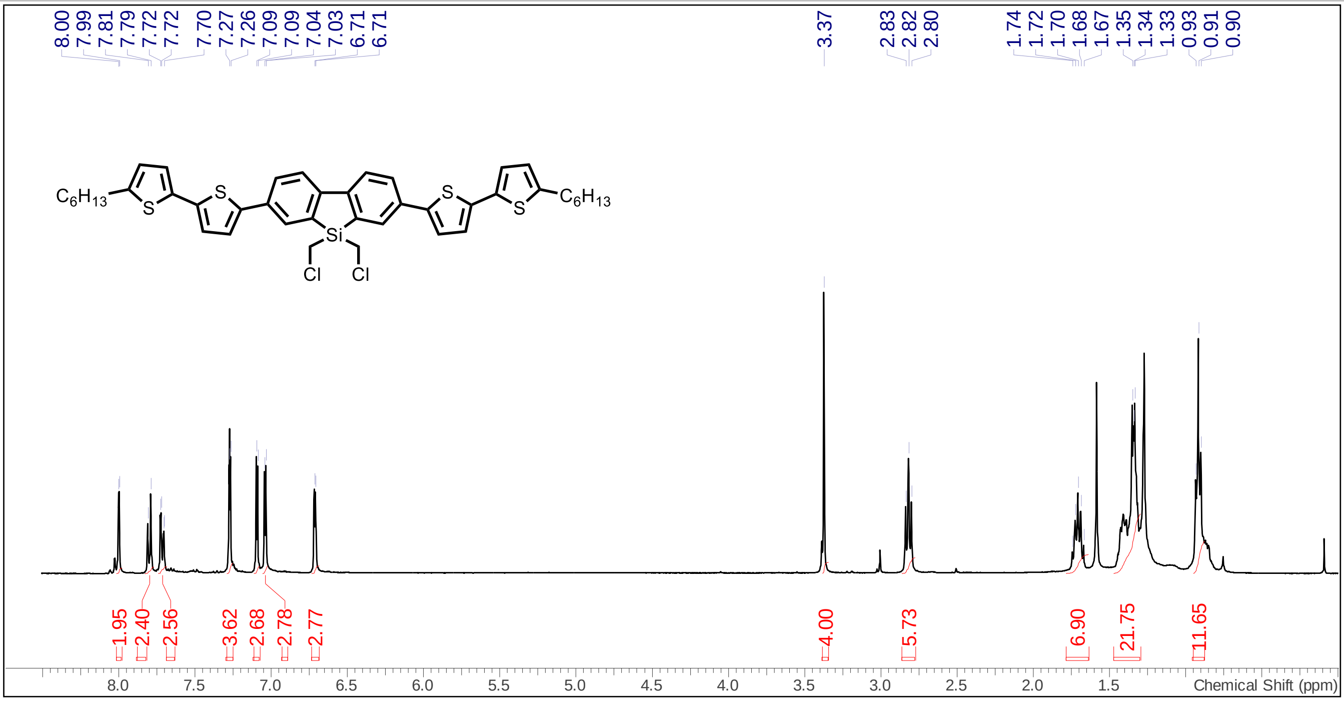

^{1}H NMR spectrum ($CDCl_3$, 400 MHz) of **4**.

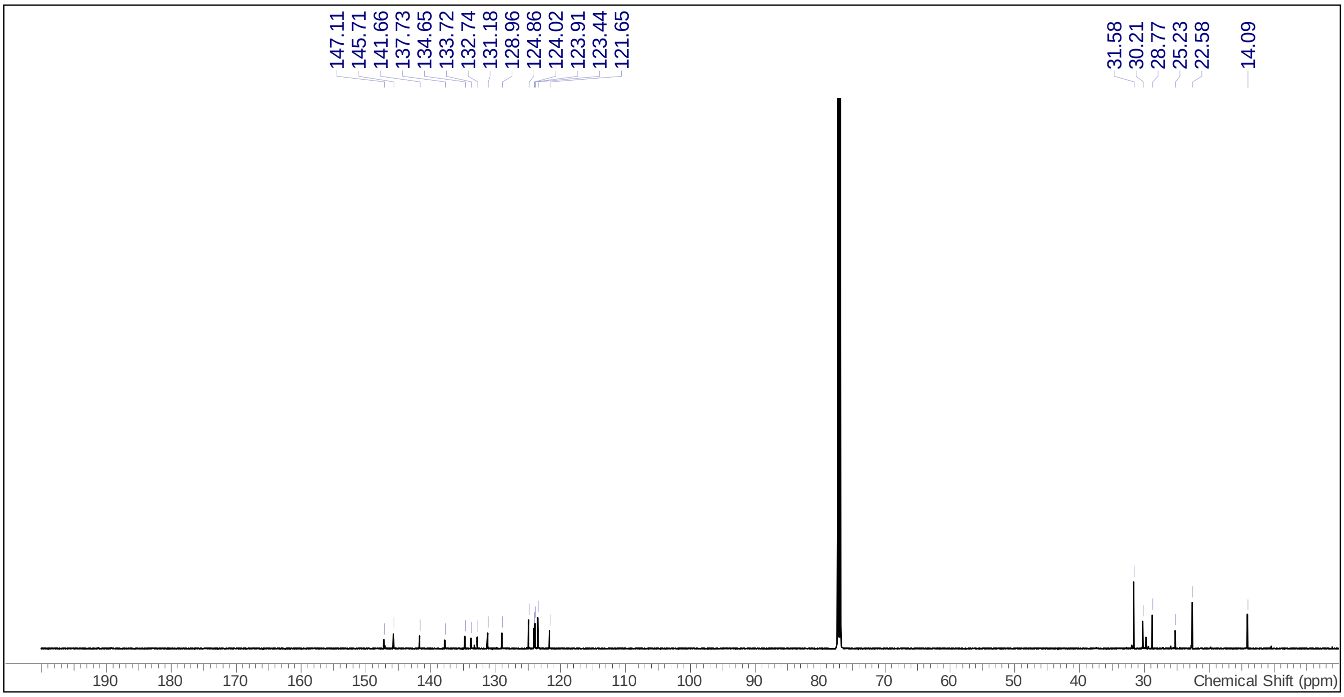

$^{13}C\{^{1}H\}$ NMR spectrum ($CDCl_3$, 151 MHz) of **4**.

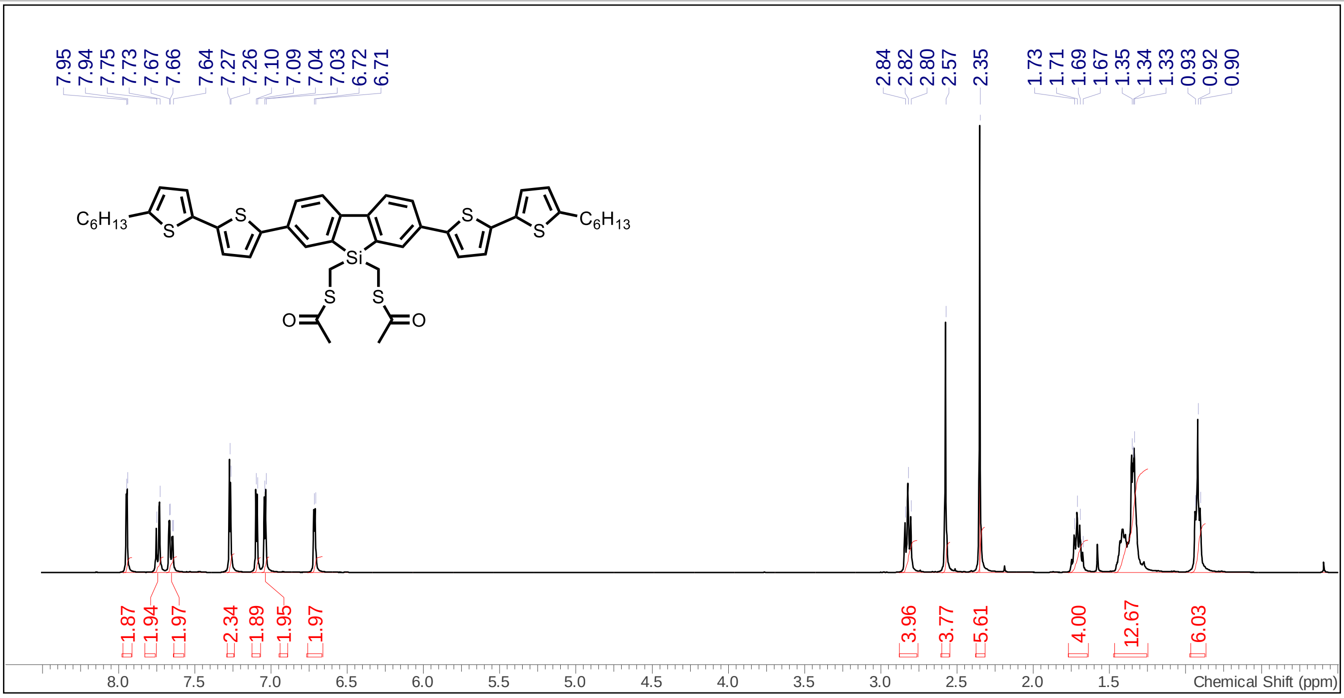

^{1}H NMR spectrum ($CDCl_3$, 400 MHz) of **5** (**PS**).

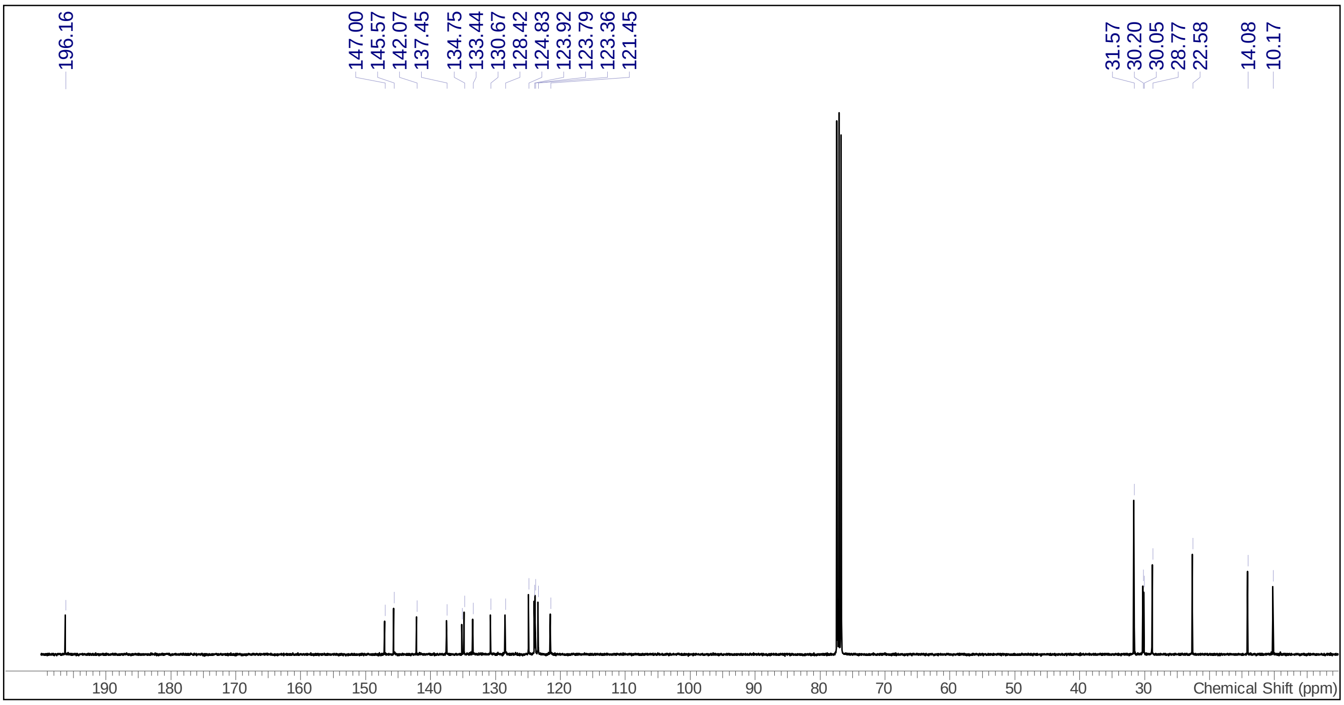

$^{13}C\{^{1}H\}$ NMR spectrum ($CDCl_3$, 101 MHz) of **5** (**PS**).

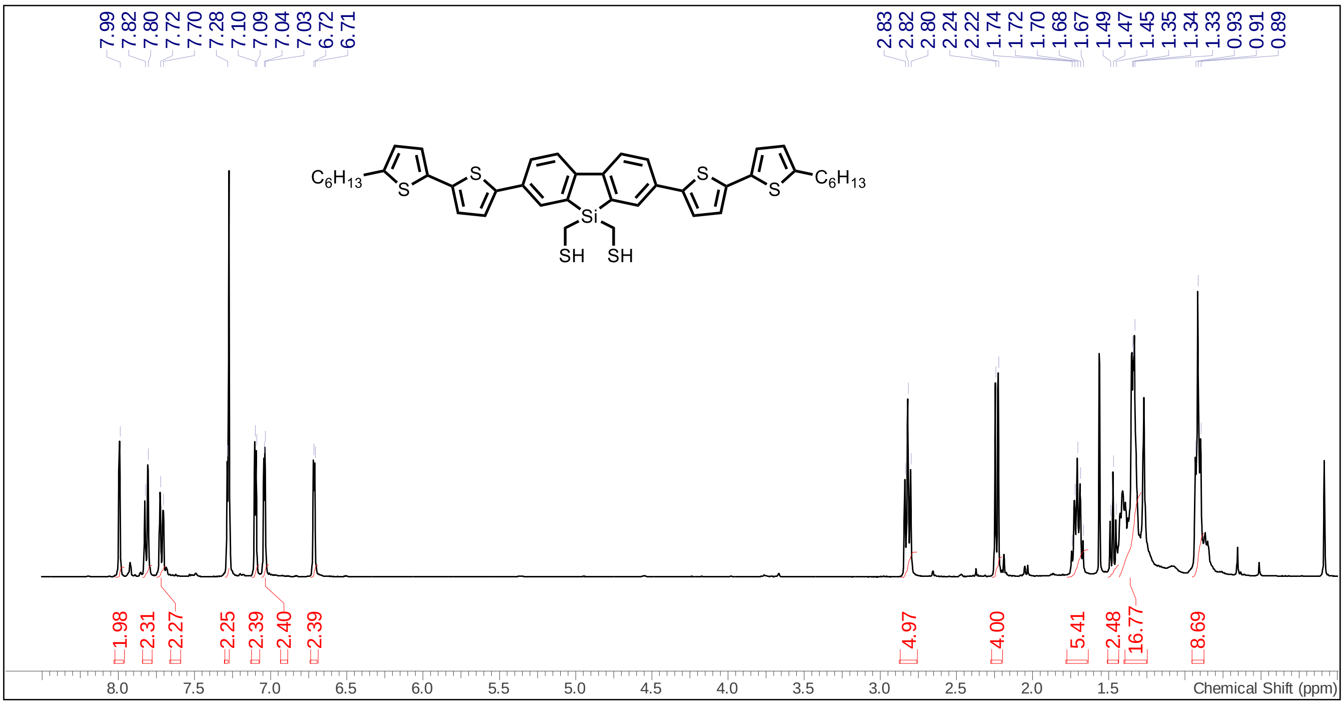

^{1}H NMR spectrum ($CDCl_3$, 400 MHz) of **6**.

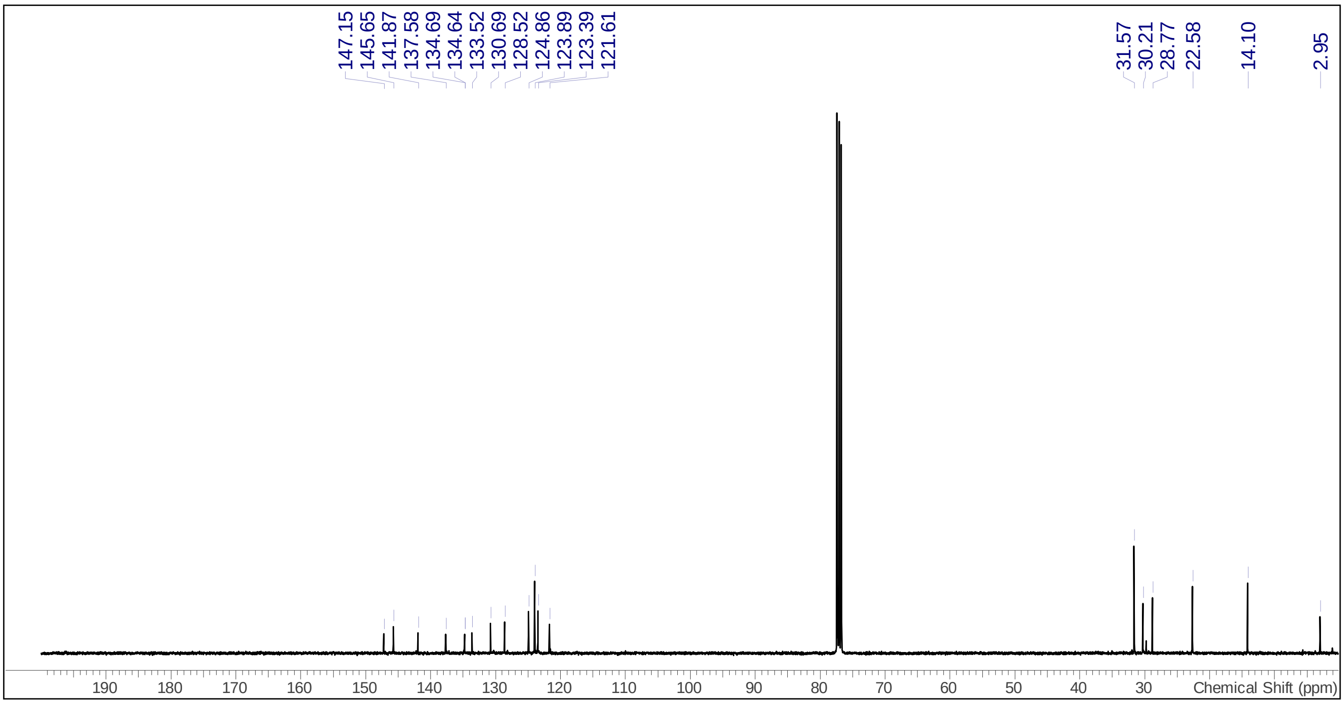

$^{13}C\{^1H\}$ NMR spectrum ($CDCl_3$, 101 MHz) of **6**.

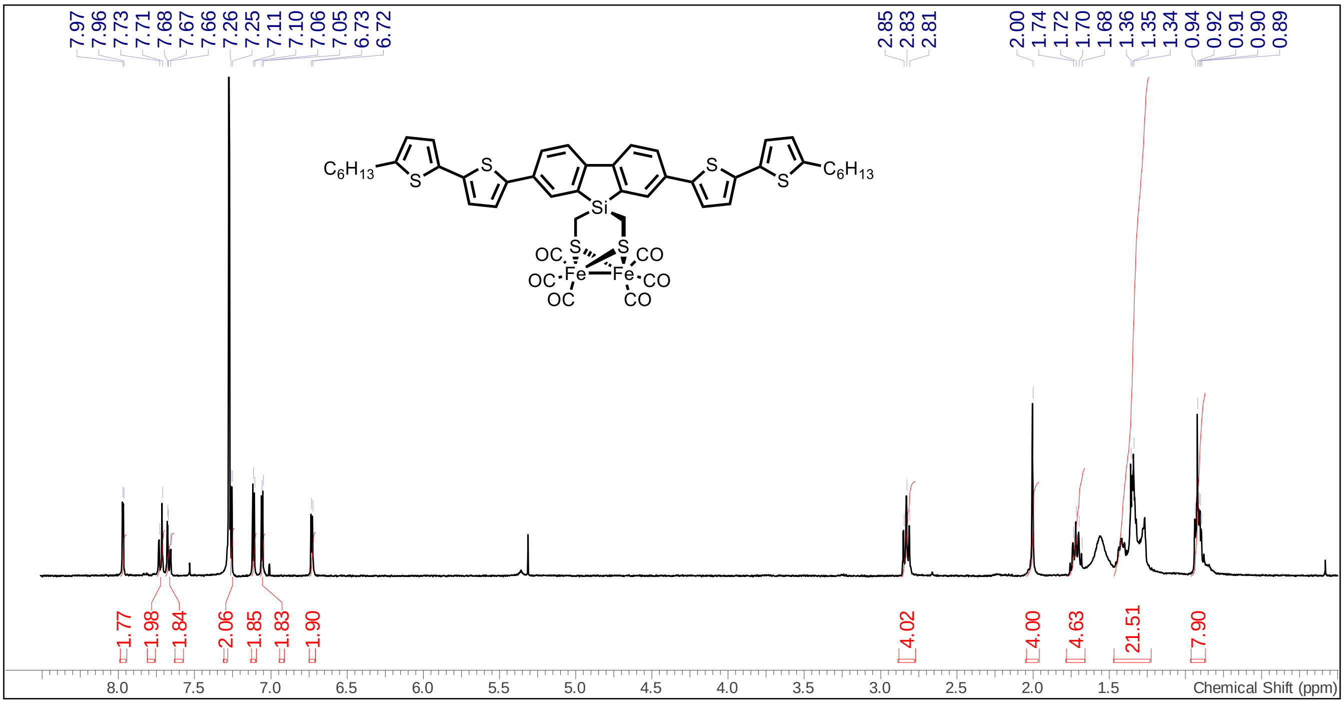

^{1}H NMR spectrum ($CDCl_3$, 400 MHz) of **7** (**PS-CAT**).

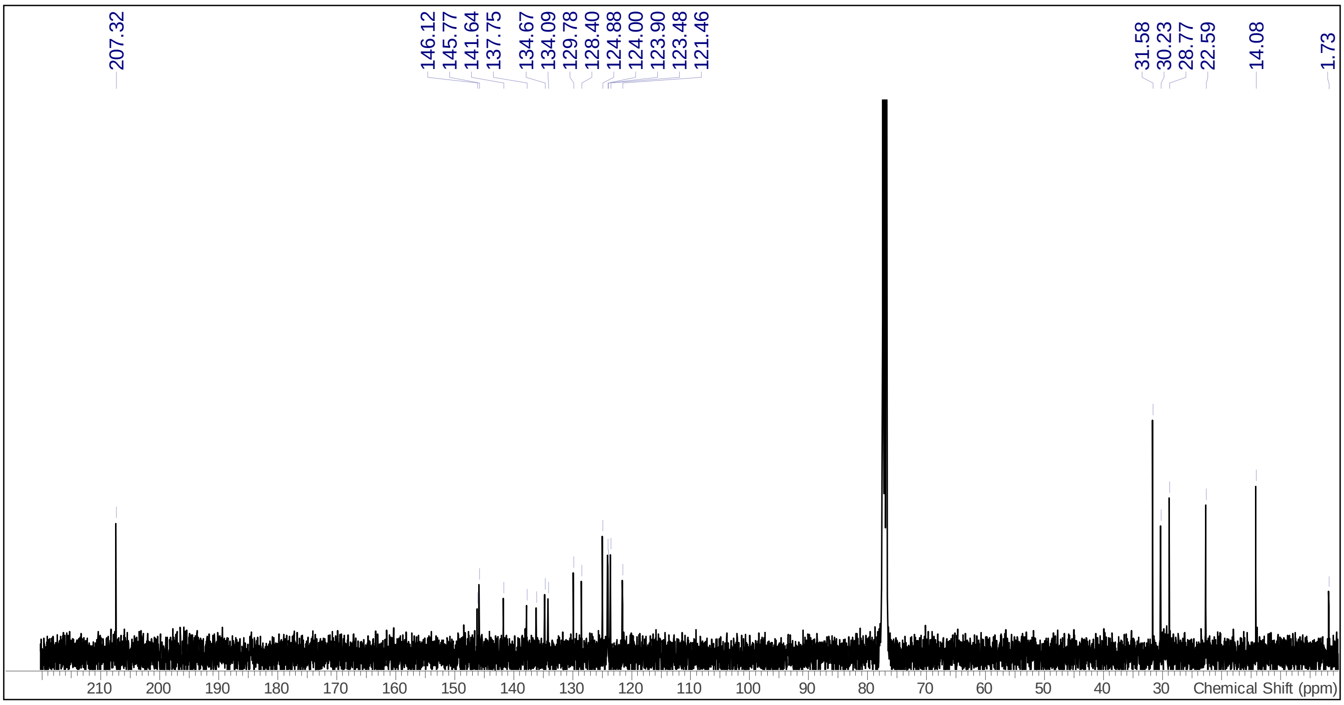

^{13}C{^{1}H} NMR spectrum ($CDCl_3$, 101 MHz) of **7** (**PS-CAT**).

Single crystal X-ray structure determination

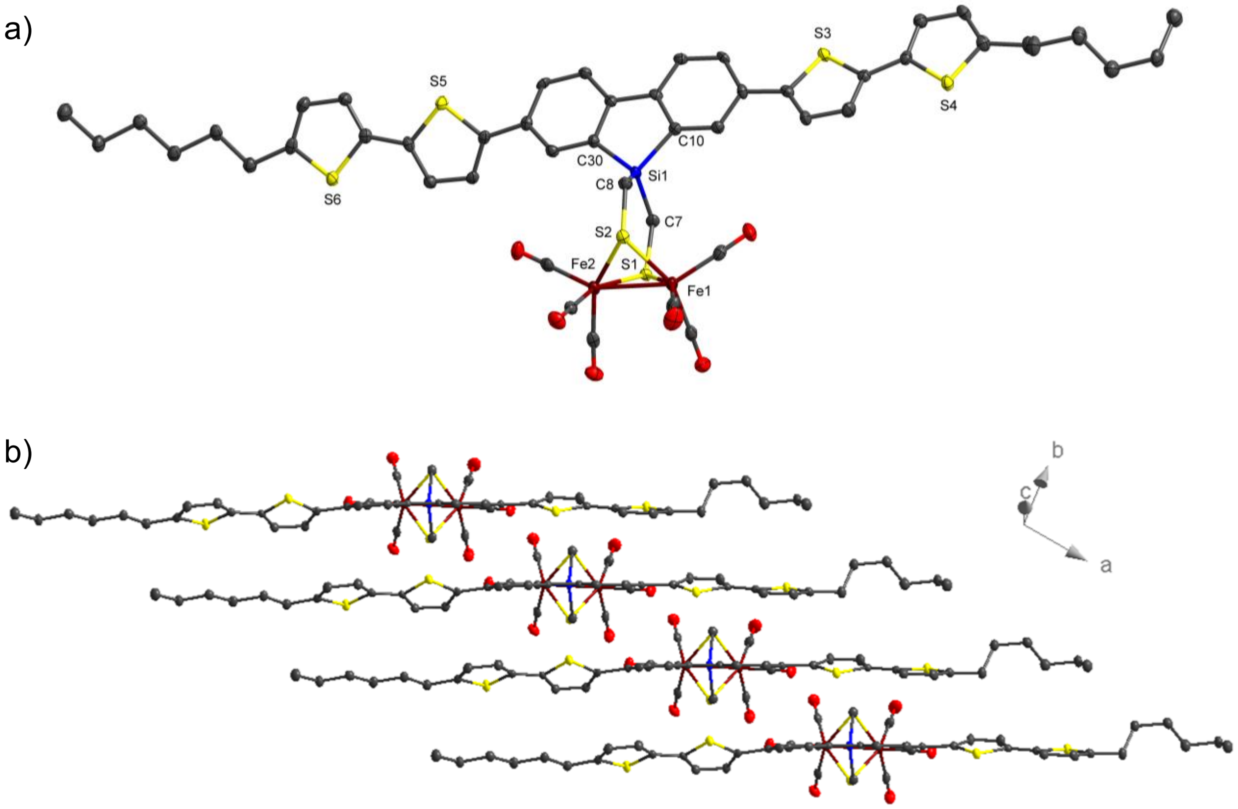

Figure S2: a) Molecular structure and atom labeling scheme of **PS-CAT**. b) View on the ab plane of stacked **PS-CAT** molecules. The stacking distance is 3.7672 Å. The ellipsoids represent a probability of 30 %, hydrogen atoms are omitted for clarity. Orange crystals suitable for X-ray structure determination were isolated from slow evaporation of a concentrated $CDCl_3$ solution.

Supporting Information available: Crystallographic data have been deposited with the Cambridge Crystallographic Data Center as supplementary publication CCDC-2106348. Copies of the data can be obtained free of charge on application to CCDC, 12 Union Road, Cambridge CB2 1EZ, UK [E-mail: deposit@ccdc.cam.ac.uk].

Table S1: Crystallographic data of **PS-CAT**.

Formula	$C_{48}H_{44}Fe_2O_6S_6Si$
fw (g·mol^{-1})	1048.98
T/K	100
crystal system	Triclinic
space group	P-1 no. 2
a/Å	7.4430(12)
b/Å	16.584(3)
c/Å	19.525(3)
α/°	102.765(2)
β/°	99.356(2)
γ/°	95.671(2)

V/Å^3	2296.4(6)
Z	2
ρ/g·cm^{-3}	1.517
μ/cm^{-1}	9.8 (MoKα)
F(000)	1084
reflections collected	17149
Θ_{max}/°	23
unique reflections (R_{int})	6208 (0.0360)
reflections with $\|F\|>4\sigma_{\|F\|}$	4907
wR_2 (all data, on $\|F\|^2$)[a]	0.0884
R_1 ($\|F\|>4\sigma_{\|F\|}$)[a]	0.0321
parameters refined	570
S[b]	1.036
max. features in final difference Fourier synthesis /e·Å^{-3}	0.426/-0.359
absorpt.corr. method	multi-scan
absorpt corr T_{min}/$_{max}$	0.977/0.990
CCDC No.	2106348

a) Definition of the R indices: $R_1 = (\Sigma || F_o| - |F_c||)/\Sigma |F_o|$;

$wR_2 = \{\Sigma[w(F_o^2-F_c^2)^2]/\Sigma[w(F_o^2)^2]\}^{1/2}$ with $w^{-1} = \sigma^2(F_o^2)+(aP)^2+bP$; $P = [2F_c^2+Max(F_O^2]/3$;

b) $S = \{\Sigma[w(F_o^2-F_c^2)^2]/(N_o-N_p)\}^{1/2}$

Table S2: Selected bond lengths and angles of **PS-CAT**.

Formation	Bond length / Å	Formation	Angle / °
Fe(1)–Fe(2)	2.5084(8)	Fe(1)–Fe(2)–S(1)	55.79(3)
Fe(1)–S(1)	2.2378(11)	Fe(1)–Fe(2)–S(2)	56.33(3)
Fe(1)–S(2)	2.2540(11)	Fe(1)–S(1)–Fe(2)	67.97(3)
Fe(2)–S(1)	2.2494(10)	Fe(1)–S(2)–Fe(2)	67.84(3)
Fe(2)–S(2)	2.2412(10)	S(1)-C(7)–Si(1)	123.19(19)
S(1)–C(7)	1.812(3)	S(2)–C(8)–Si(1)	123.2(2)
S(2)–C(8)	1.815(3)	C(7)–Si(1)–C(8)	112.29(16)
C(7)–Si(1)	1.865(3)	C(10)–Si(1)–C(30)	91.47(15)
C(8)–Si(1)	1.873(4)	C(7)-Si(1)–C(10)	110.68(15)
Si(1)–C(10)	1.857(3)	C(8)–Si(1)–C(30)	115.38(16)

Si(1)–C(30)	1.866(4)	C(10)–Si(1)–C(8)	111.70(15)
		C(30)–Si(1)–C(7)	113.59(16)

Photocatalysis

Irradiation experiments were executed in LABSOLUTE clear glass screw neck vials (ND13) and screw seals (ND13, butyl red / PTFE grey) (total volume 5 mL) filled with 10 µM **PS-CAT** complex solutions with 1000 equivalents of BIH as sacrificial donor in CH_3CN/NMP (5:1). The samples were prepared under a nitrogen atmosphere to exclude any oxygen, solvents were deaerated thoroughly with an argon flow. Additionally, the vials were sealed with parafilm. Hydrogen quantification was carried out from each separate vials taking 200 µL headspace volume.

Detailed results

Photocatalytic series were started with a M420LX Mounted LED from Thorlabs due to an appropriate overlapping between absorption (**PS-CAT**) and emission spectra (LED). However, a red-shifted M455L3 Mounted LED could even improve the photocatalytic performance (420 nm: TON = 72 after 16 h; 455 nm: TON = 145 after 17 h), although the spectral overlapping is less. The difference could also arise from a different LED power (420 nm: ≈ 7 mW cm^{-2}, photon density ≈ 2.5E-8 mol photons s^{-1} cm^{-2}; 455 nm: ≈ 10 mW cm^{-2}, photon density ≈ 3.8E-8 mol photons s^{-1} cm^{-2}; vial area: 1.23 cm^2), respectively.

Initially ($\lambda_{em,LED}$ = 420 nm), the experiments were carried out in a solvent mixture of CH_3CN/THF (5:1). Replacement of THF by NMP significantly increased the catalytic turnover, *e.g.*, after 16 hours of irradiation: TON ≈ 5 from CH_3CN/THF (5:1) solution, TON = 72 from CH_3CN/NMP (5:1) solution. This can be explained by the basic properties of NMP, capturing the proton of the oxidized donor ($BIH^{\bullet+}$) and thus inhibiting potential back electron transfer processes.[11]

According to their redox potential, even other electron donors should be suitable to drive HER under light, as (TD)DFT calculations revealed an energy gap between (T_3|D_0) (**PS-CAT**) and (D_0|S_0) (Fc^+/Fc) of $\Delta\Delta E$ = -0.45 eV (Table S7), which in principle means, that sacrificial donors with a redox potential less positive than 0.45 V should be capable of reducing the system and to drive HER. However, as shown in Table S3, several experiments with potential donors, partly in combination with an additional proton source, yielded in very low catalytic rates after a moderate irradiation ($\lambda_{em,LED}$ = 420 nm) time. It should be noted that the given redox potentials are roughly recalculated against Fc^+/Fc from original data (for details see Table S3). What also contributes to the imprecision of the listed values, is the relation to an aqueous (ascorbic acid: HAsc, triethanolamine: TEOA) or pure CH_3CN solution (benzyl mercaptan: BnSH, 1,3-dimethyl-2-phenylbenzimidazoline: BIH, 1-Benzyl-1,4-dihydronicotinamide: BNAH).[12]

Table S3: Results of selected photocatalytic experiments of 10 µM **PS-CAT** with common electron donors and $\lambda_{em,LED}$ = 420 nm. Redox potentials are roughly recalculated against Fc^+/Fc from original data by subtracting -0.63 V[a][13] (published *vs.* NHE) or -0.38 V[b][13] (published *vs.* SCE).[12]

Electron donor	Proton donor	≈ *E vs.* Fc^+/Fc < 0.45 V	Solvent	Time	TON
HAsc<br>1000/10000 eq<br>5000 eq		0.08 V[a]	 THF/H_2O (20:1)<br>NMP	 12 h<br>23 h	 < 1<br>< 1
TEOA<br>10000 eq	TFA<br>5000 eq	0.19-0.44 V[a]	 CH_3CN/THF (3:1)	 16 h	 < 1
BnSH<br>1000/10000 eq		-0.48 V[b]	 CH_3CN/THF (3:1)	 15.5 h	 < 1

BnSH<br>10000 eq	TFA<br>5000 eq	-0.48 V[b]	CH_3CN/THF (3:1)	16 h	< 1
BIH<br>1000 eq<br>1000 eq<br>1000 eq		-0.05 V[b]	 CH_3CN/THF (3:1)<br>CH_3CN/THF (5:1)<br>CH_3CN/NMP (5:1)	 16.5 h<br>16 h<br>16 h	 ≈ 2<br>≈ 5<br>≈ 72
BNAH<br>1000 eq		0.19 V[b]	CH_3CN/NMP (5:1)	16 h	< 1

UV-vis-monitored photocatalysis

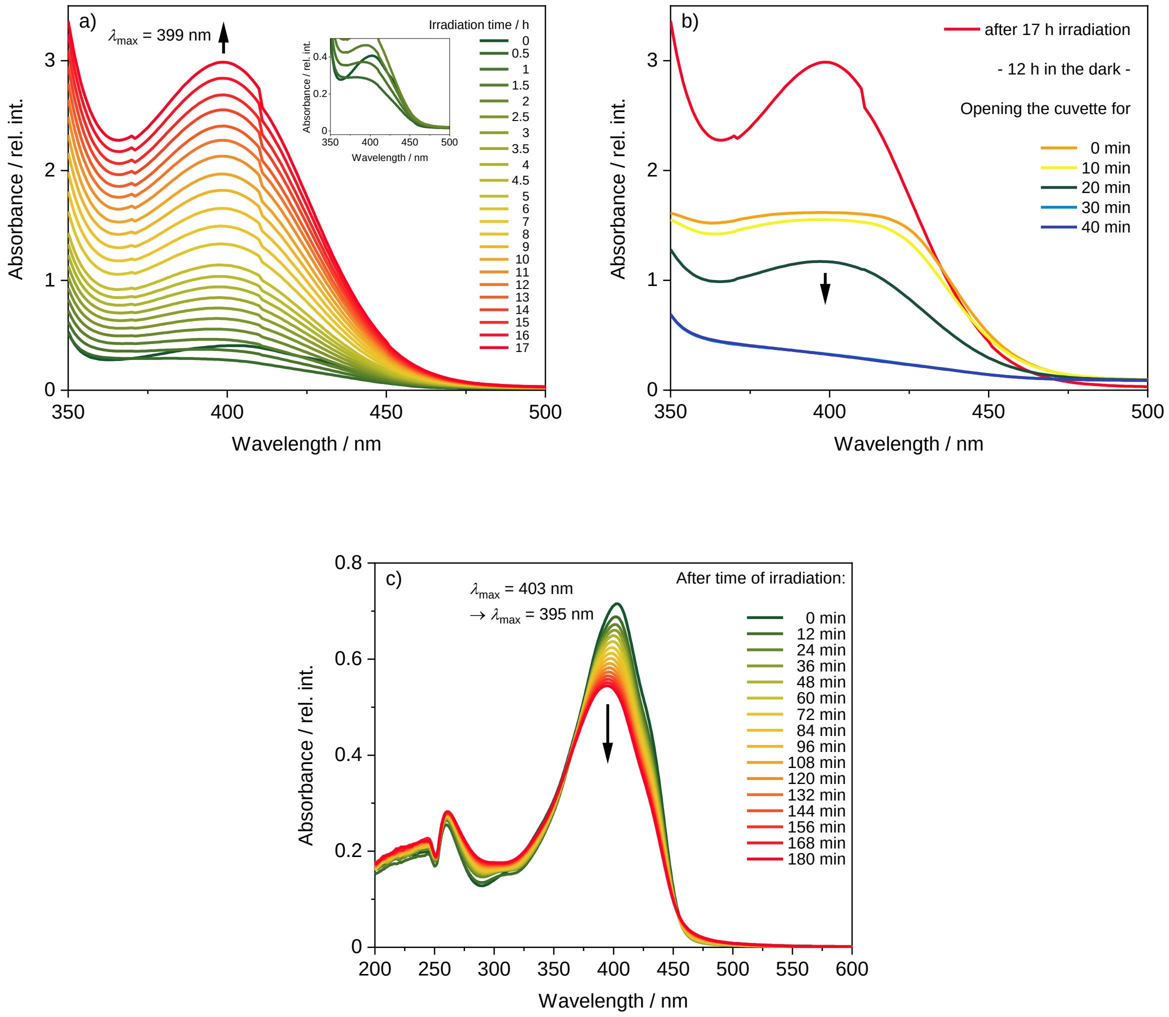

Figure S3: a) UV-vis spectroscopic monitoring of a photocatalytic experiment (10 µM **PS-CAT** and 1000 eq BIH in CH_3CN/NMP = 5:1, V = 1 mL) over a period of 17 h. During the UV-vis measurement the stirrer and the LED light (455 nm) were turned off. b) UV-vis spectroscopic monitoring of the dark and oxygen quenching process for the newly formed species. c) UV-vis spectroscopic monitoring of the photostability of **PS-CAT** in CH_3CN/NMP = 5:1 upon irradiation (455 nm). All measurements were carried out in a 1 cm x 0.5 cm inert cuvette. After irradiation of the narrow cuvette side (LED power ≈ 180 mW), the UV-vis measurement was executed throughout the wide cuvette side.

Fluorescence quenching in presence of BIH

Emission quenching experiments were performed to investigate potential interaction between **PS** and **PS-CAT** with the quencher BIH. The quenching experiments were performed by adding increasing equivalents of BIH to a solution of **PS** and **PS-CAT** in a titration experiment. Not only changes in emission intensity (and spectral shape of the emission spectra) have been followed, but also in parallel absorption spectra of the solutions with increasing BIH concentration were collected during this experiment.

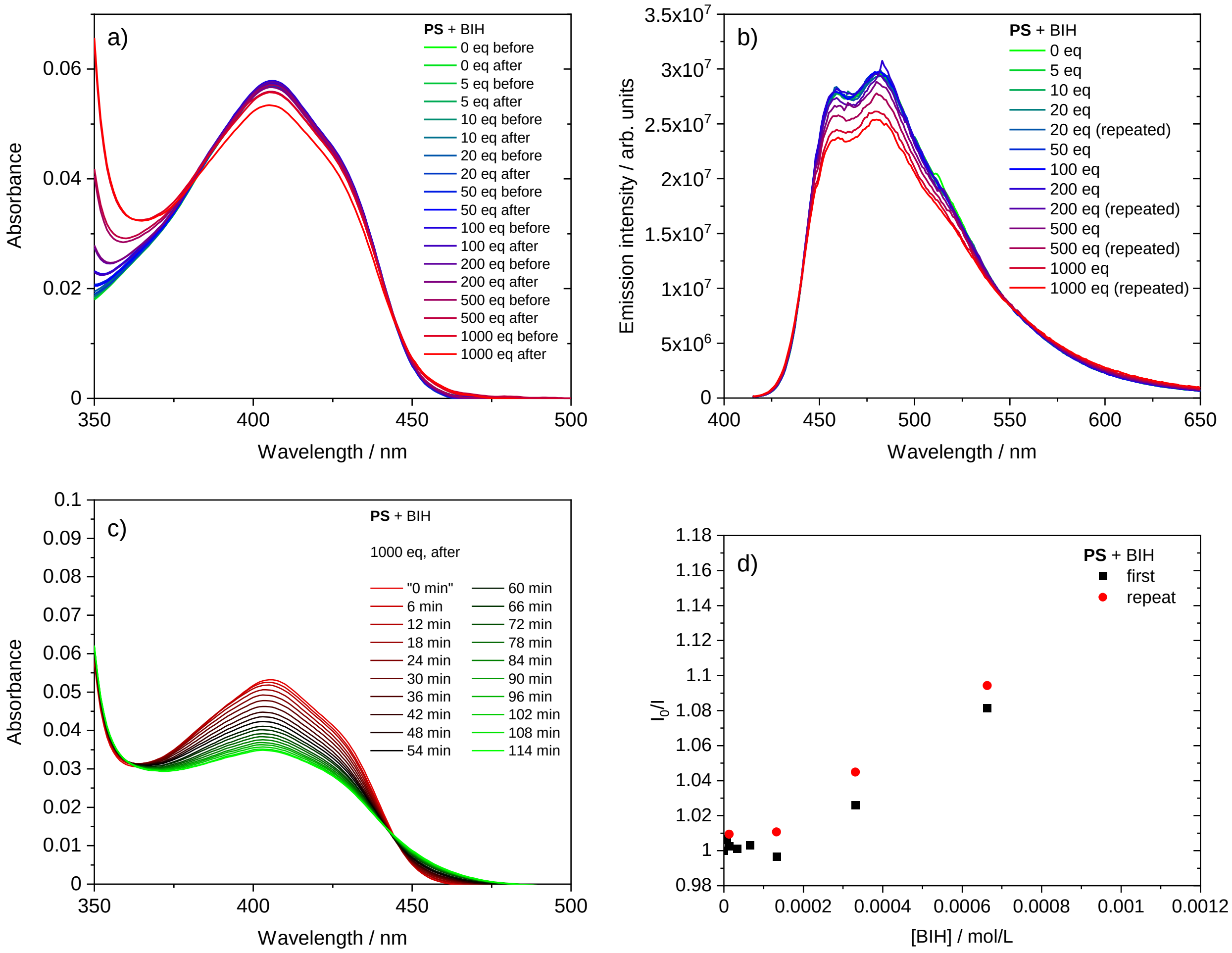

Figure S4: a) Absorption spectra of **PS** (c = $6.63 \cdot 10^{-7}$ mol/L) and b) emission spectra of **PS** upon addition of equivalents of BIH in dry and deaerated acetonitrile. All spectra are corrected for dilution effects occurring upon addition of increasing amounts of BIH. Absorption spectra were recorded for each concentration before and after the respective emission measurements to observe potential changes in the absorption spectrum during this time. For chosen concentrations several emission spectra were recorded sequentially to test for changes in emission intensity with time. c) Development of absorption spectra after addition of BIH. d) Respective plot of I_0/I *vs.* the concentration of BIH illustrating the changes in emission intensity in the presence of increasing amounts of BIH. For selected concentrations several sequential emission spectra were recorded. The repeated measurements after few minutes are represented by red circles.

For **PS** we noticed that in the solvent mixture (CH_3CN/NMP = 5:1) applied in the catalytic experiment, **PS** apparently is lacking necessary stability, which shows in a decrease of absorbance and decreasing emission intensity with time, even without addition of BIH or additional irradiation. This behaviour is induced by the presence of NMP and the experiments for **PS** were performed in pure acetonitrile, where **PS** was stable. Upon addition of BIH, at low equivalents, we observe negligible quenching up to 200 equivalents. Only in the presence of 500 and 1000 equivalents the emission intensity of **PS** is significantly decreased, and the extent of quenching increases with time

as shown by sequentially collected emission spectra at the same concentration. This correlates with slight changes in the absorption spectra which we recorded in parallel. A decrease of the absorption band with time after addition of BIH can be observed. In our series of experiments, we recorded absorption spectra before and after each emission measurement at a certain BIH concentration and we performed an additional experiment upon addition of 1000 equivalents following the development of a time range of longer than 100 minutes. After this time scale the absorption spectra is changed significantly, represented by a loss of absorbance of the visible absorption band. Whether this effect is caused by an aggregation effect or by a reaction between **PS** and BIH, which is changing the chromophore, is not clear at the current state of research. Under these conditions an evaluation of the quenching experiment following classical Stern-Volmer formalism is not possible.

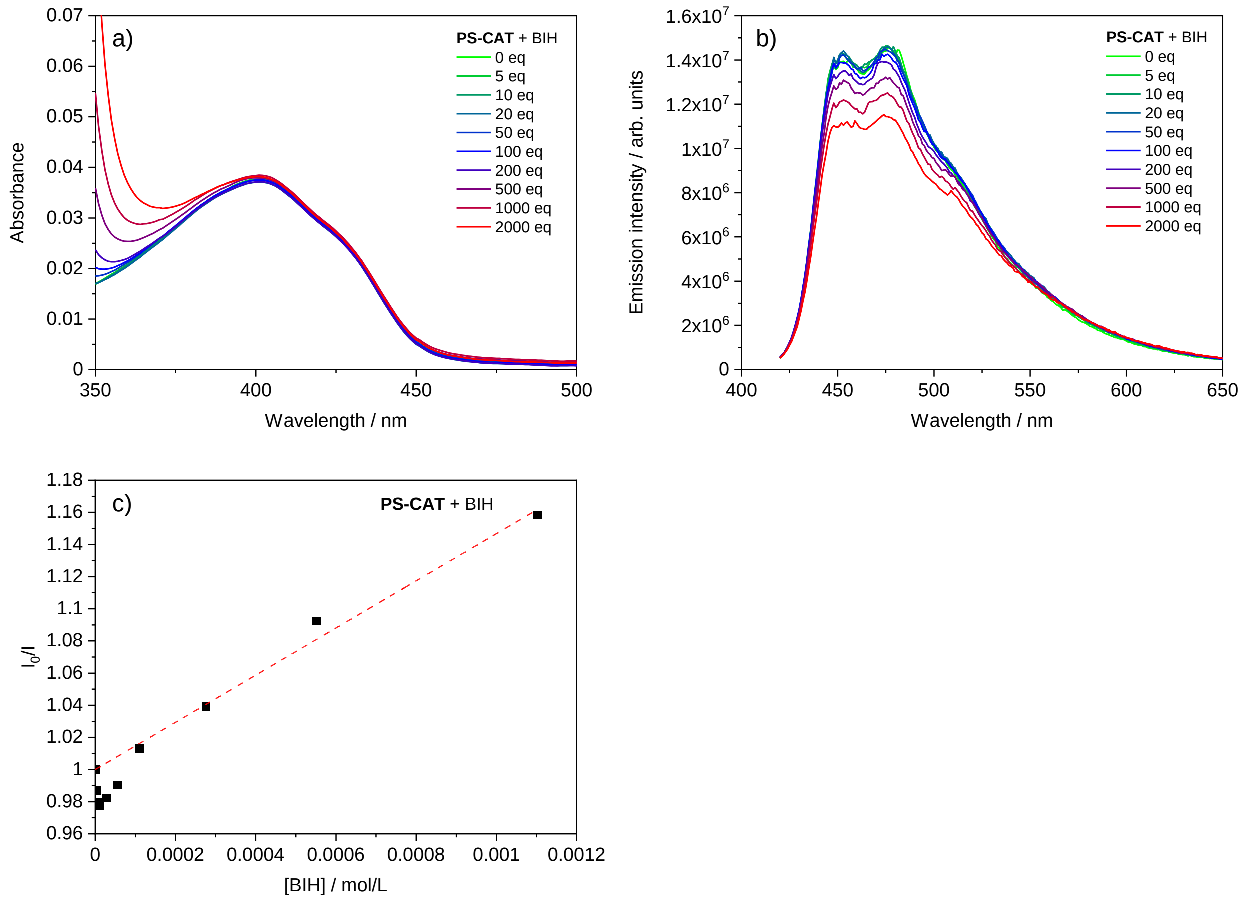

Figure S5: a) Absorption spectra of **PS-CAT** (c = 5.51·10^{-7} mol/L) and b) emission spectra of **PS-CAT** upon addition of equivalents of BIH in a dry and deaerated mixture of CH_3CN/NMP = 5:1. All spectra are corrected for dilution effects occurring upon addition of increasing amounts of BIH. c) Respective plot of I_0/I *vs.* the concentration of BIH illustrating the changes in emission intensity in the presence of increasing amounts of BIH. The red dashed line is the respective Stern-Volmer fit.

In analogy to the investigations for **PS**, quenching experiments were also performed by adding BIH to a solution of **PS-CAT** with parallel monitoring of the absorption spectra of the solutions with increasing amount of BIH. These experiments were performed in a mixture of CH_3CN/NMP = 5:1. For **PS-CAT** the presence of NMP was essential to gain solubility and the complex is stable under these conditions. In contrast to **PS**, **PS-CAT** does not show any changes in the absorption spectra under these conditions during the quenching experiment, which means the processes leading to the changes in absorbance for **PS** in presence of BIH are not occurring for **PS-CAT**. The emission intensity decreases noticeably. A Stern-Volmer analysis was performed in this case. For collisional quenching the following relation applies:

$$\frac{I_0}{I} = 1 + K_D[Q] = 1 + k_q\tau[Q]$$

with I_0 the integral intensity of the emission band in absence of quencher, I the integral intensity in presence of quencher, K_D the dynamic Stern-Volmer constant [L/mol], [Q] the quencher concentration [mol/L], k_q the bimolecular quenching rate constant [L/mol s], τ lifetime of the fluorophore in absence of the quencher [s].

For static quenching with complex formation

$$\frac{I_0}{I} = 1 + K_S[Q]$$

with K_S the static Stern-Volmer constant [L/mol] or association constant applies.

For a system following the Stern-Volmer relation (independent of dynamic or static quenching) plotting I_0/I *vs.* [Q] results in a linear relation.

The fit reveals a Stern-Volmer constant of 146 L/mol, which results with a lifetime of the emission of 1 ns a bimolecular quenching constant of $1.5 \cdot 10^{11}$ L/mol s. This is larger than the typical range for diffusion limited processes and can be an indication for a static quenching process playing a role, which in principle enables ultrafast quenching.

Time-resolved spectroscopy

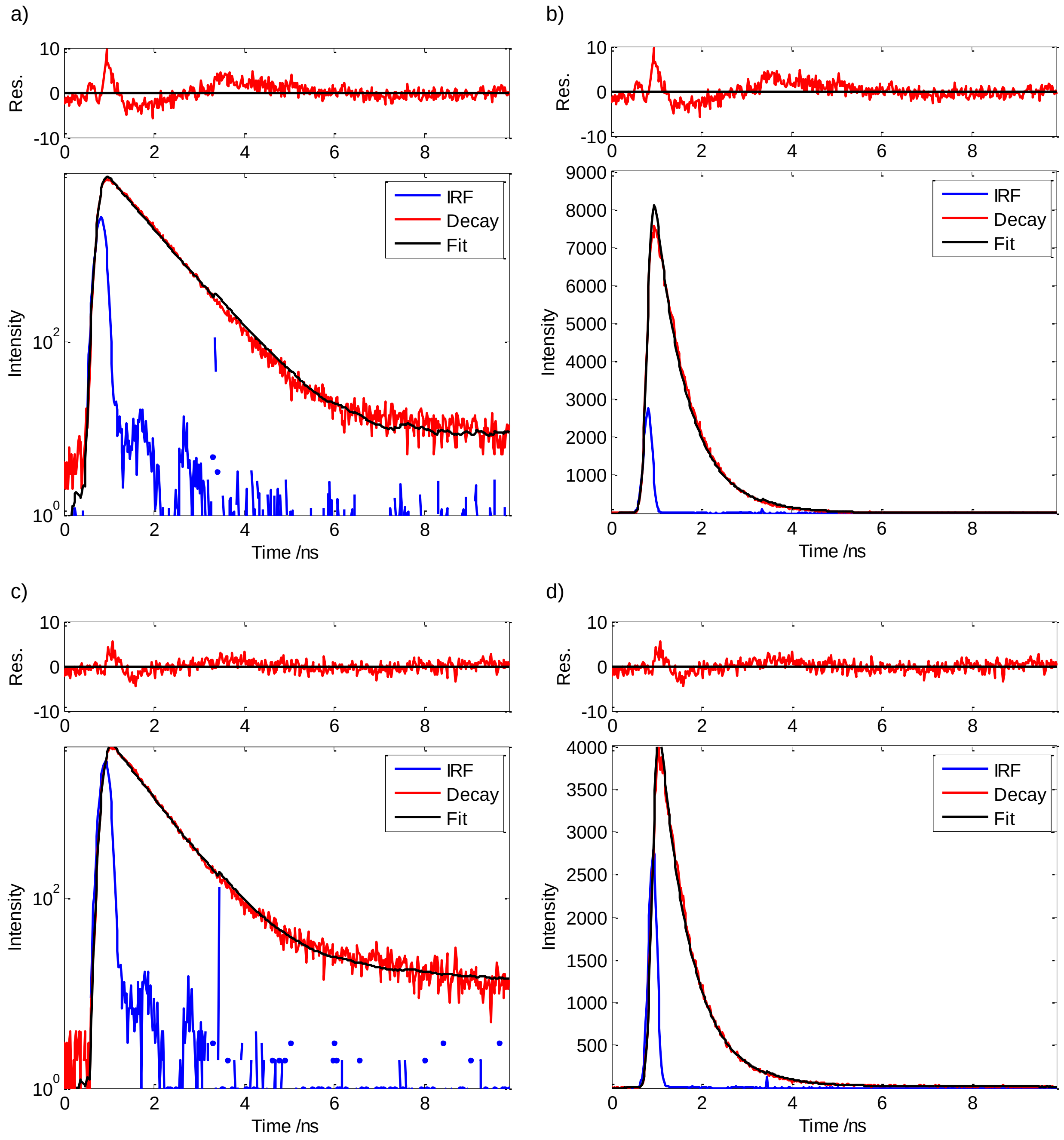

Figure S6: Streak camera measurements of **PS** (a, b) and **PS-CAT** (c, d). a, c) Trace on the regular scale. b, d) Trace on the logarithmic scale. The samples were prepared to yield an optical density of 0.1 at the excitation wavelength in a 1.0 cm inert quartz cuvette using deaerated, spectroscopic grade THF.

Emission lifetimes were determined for **PS** (τ_1 = 1 ns) and **PS-CAT** (τ_1 = 1 ns) in deaerated THF at an optical density of about 0.1. The absence of emission lifetime changes indicates that a potential quenching process leading to decreased emission quantum yields occurs on the ultrafast timescale or quasi instantaneously upon excitation, *e.g. via* partial population of the charge-separated singlet state (S_2) predicted by TDDFT and cannot be resolved in the emission lifetime measurements due to limited temporal resolution.

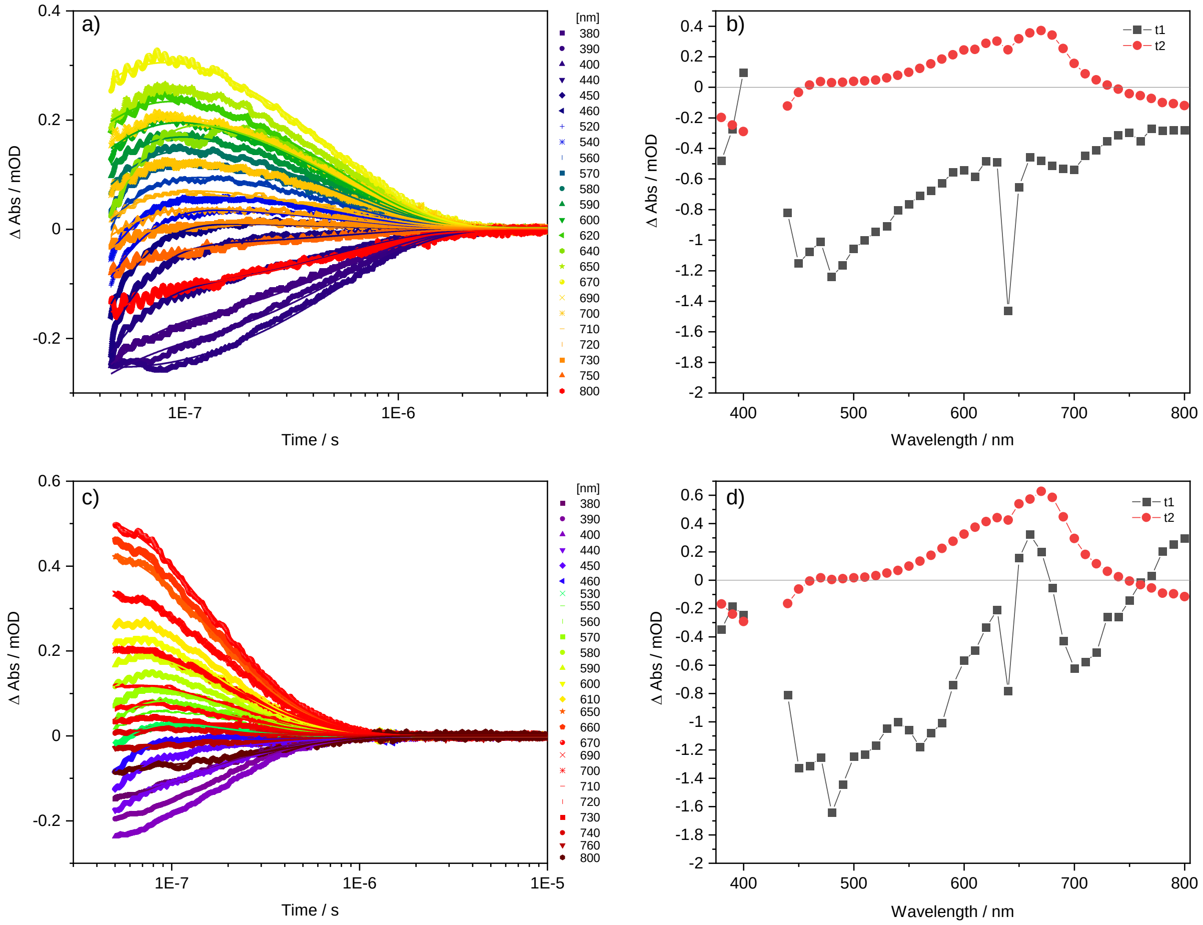

Figure S7: Pulsed ns-TA measurements of **PS** (a, b) and **PS-CAT** (c, d). a, c) Selected kinetic traces with corresponding biexponential fit. b, d) Decay associated spectra resulted from the global fit of the kinetic data. The samples were measured in 1 cm path length cuvettes at an optical density of around 0.25 at the excitation wavelength.

EPR spectroscopy

EPR spectroscopy was carried out under catalytic conditions with a **PS-CAT** concentration of 30 µM to increase the concentration of the paramagnetic species. Samples were prepared under inert gas in the glovebox by solving **PS-CAT** in NMP and CH_3CN and adding 1000 equivalents of BIH. The solution was filled into an EPR tube with 3 mm diameter. Illumination was carried out at 270 K for 4 hours in the resonator, afterwards the system was cooled down to 4 K for the measurement. Measurement conditions – Microwave frequency: 9.387026 GHz, modulation amplitude: 0.2 mT, microwave power: 1.5 mW, 4 scans.

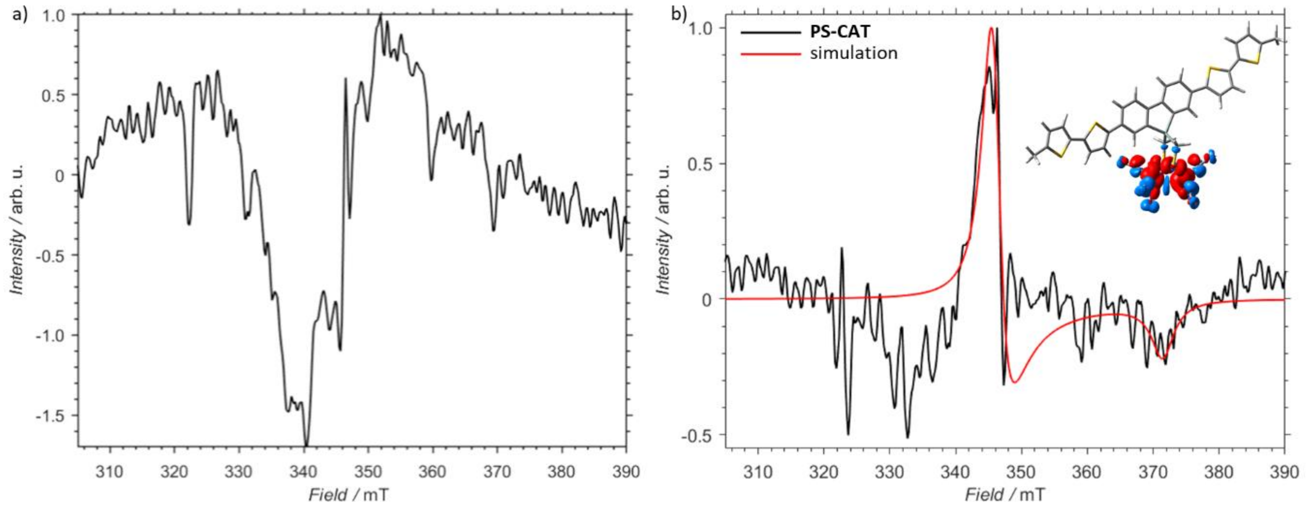

Figure S8: a) **PS-CAT** before illumination of the sample at 4 K. Dip at 340 mT stems from the resonator. b) **PS-CAT** after 4 hours illumination. Baseline is subtracted for better visibility of the peak; spin density localized at the [FeFe] moiety of the singly reduced **PS-CAT** (doublet).

Electrochemical characterization

CV was carried out in dry and deaerated 0.1 M CH_2Cl_2-[*n*-NBu_4][BF_4] solutions with a complex concentration of 1 mM. The working electrode was polished with alumina after each measurement. Oxygen-free environment in the measuring cell was realized by purging both headspace (permanently) and solution (during opening the cell) with a nitrogen flow. The ferrocenium/ferrocene (Fc^+/Fc) couple served as reference.

PS-CAT shows a (quasi-)reversible reduction wave at $E_{1/2}^{Red1}$ = -1.61 V in CH_2Cl_2 (Figure S9a), which is assigned to the reduction of [Fe^IFe^I] to [Fe^IFe^0] and similar to previous reported complexes in this solvent.[10, 14] The current function I_{pc} / ($cv^{1/2}$) for different scan rates ($0.2 \le v \le 20$ Vs^{-1}) (Figure S9b)) reveals a one-electron transfer for this process, based on a comparison between a one- [(pdt)$Fe_2(CO)_6$][15] (pdt = propane-1,3-dithiolate) and a two-electron [(bdt)$Fe_2(CO)_6$][16] (bdt = benzo-1,3-dithiolate) transfer reference. This is underlined by the observed vibrational shift of the original *v*(CO) (2072, 2033, 2000 and 1988 cm^{-1}) upon electrochemical CAT reduction (Figure S10). Here, *i.e.*, the IR bands at 2072 and 2033 cm^{-1} presumably are shifted to 2020 and 1970 cm^{-1}, which is of a similar order of magnitude as for the one-electron reference [(pdt)$Fe_2(CO)_6$] (shift by ca. 70 cm^{-1} upon electrochemical reduction).[15] As opposed to this, the two-electron reference [(bdt)$Fe_2(CO)_6$] shows shifts in the region of 110-140 cm^{-1}, respectively.[16]

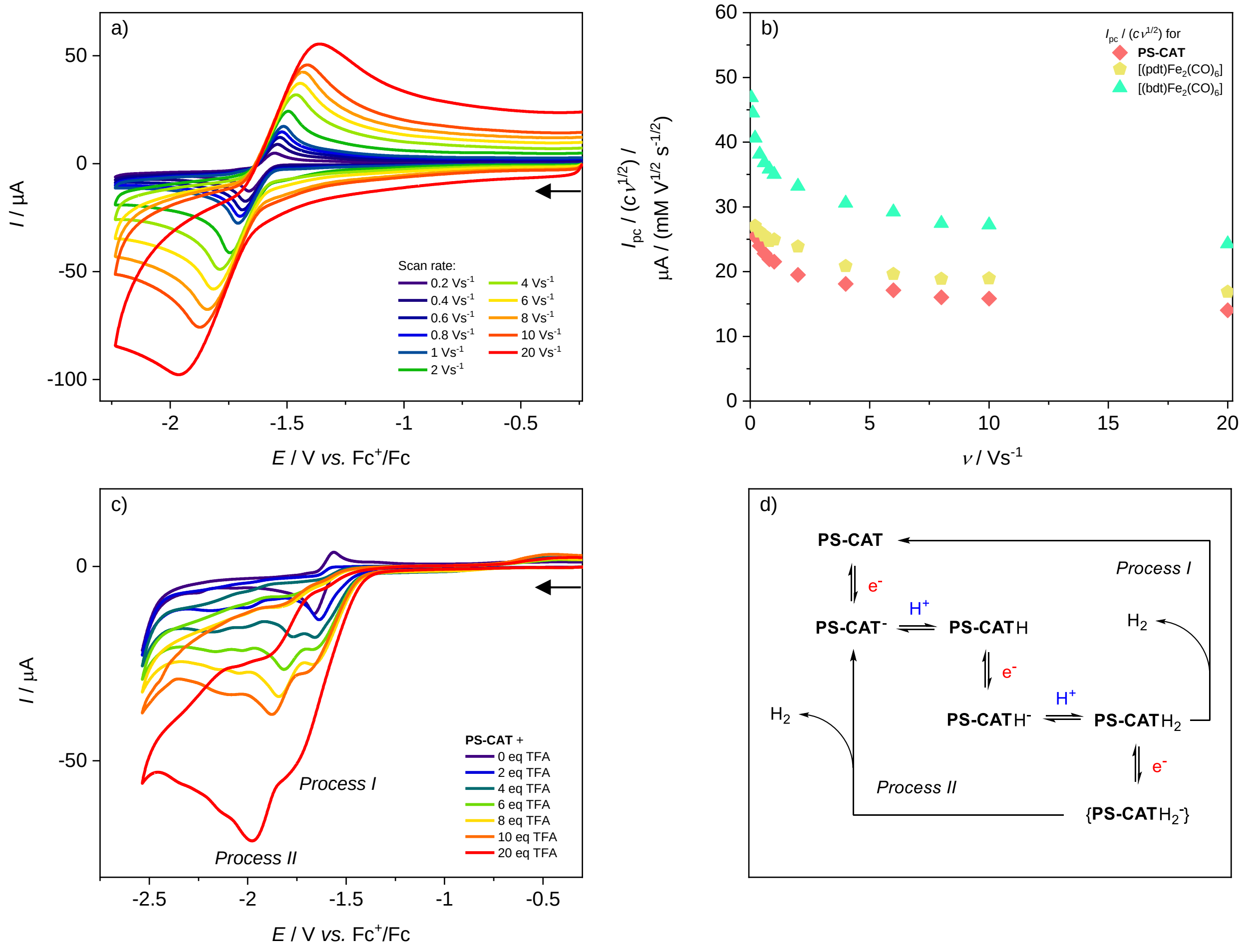

Figure S9: a) Cyclic voltammograms of 1 mM **PS-CAT** in 0.1 M CH_2Cl_2-[*n*-NBu_4][BF_4] at different scan rates. b) Corresponding current function of **PS-CAT**, compared with these for [(pdt)$Fe_2(CO)_6$] and [(bdt)$Fe_2(CO)_6$]. c) Cyclic voltammograms of 1 mM

PS-CAT in 0.1 M CH_2Cl_2-[*n*-NBu_4][BF_4] at a scan rate of 0.2 Vs^{-1} and different amounts of TFA. d) Proposed mechanism for the electrochemical proton reduction cycle of **PS-CAT** with TFA. Arrows indicate the scan direction.

The electrocatalytic property of **PS-CAT** was investigated by the addition of various amounts of trifluoroacetic acid (TFA) (Figure S9c), since only strong acids are capable of protonating a monoanionic species (one-electron transfer).[15] Upon addition of acid, the reduction potential of [Fe^IFe^I] to [Fe^IFe^0] is shifted from E_{pc} = -1.66 V (without acid, formation of **PS-CAT**$^-$) to -1.64 V (two equivalents of TFA) due to the follow-up protonation and generation of **PS-CAT**H. For higher acid amounts (up to 20 equivalents of TFA) the current for this reduction is increasing, which exposes a first catalytic process (process I). Likewise, another catalytic reduction process (process II) emerges at four equivalents of TFA (E_{pc} = -1.77 V, shifts to -1.98 V at 20 equivalents of TFA) and becomes more and more dominant up to 20 equivalents of TFA. Based on the behaviour of [(pdt)$Fe_2(CO)_6$] towards a strong acid (TfOH),[15] **PS-CAT** could follow a similar proton reduction cycle (Figure S9d).

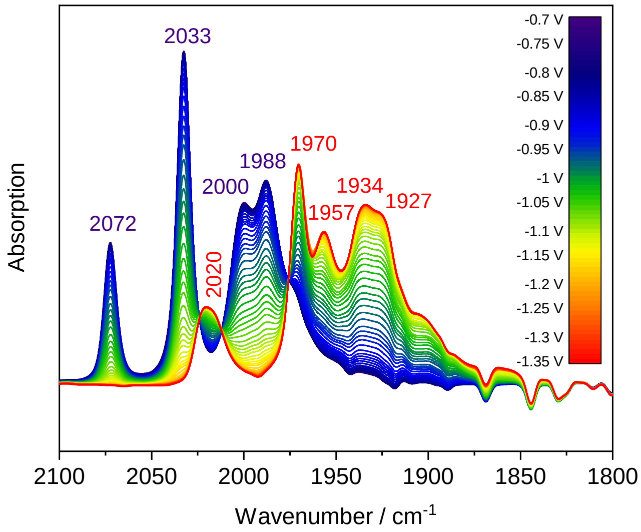

Figure S10: IR SEC of 2 mM **PS-CAT** (THF) during reduction.

UV-vis SEC (Figure S11/S12) was achieved by using a UV-vis SEC cuvette with a 1 mm pathway equipped with a glassy carbon electrode as the working electrode, platinum wire as the counter electrode and silver wire as the reference electrode. The glassy carbon electrode has a hole in the center of the measurement window to ensure a high concentration of oxidized and reduced species is available for the measurement. The setup was degassed by a constant N_2 flow 15 min before solution was added and during the measurements. Both **PS** and **PS-CAT** were solved in THF at a concentration of $2 \cdot 10^{-4}$ M with 0.1 M [*n*-NBu_4][BF_4] as the electrolyte. Oxidation and reduction were carried out with fresh solutions respectively.

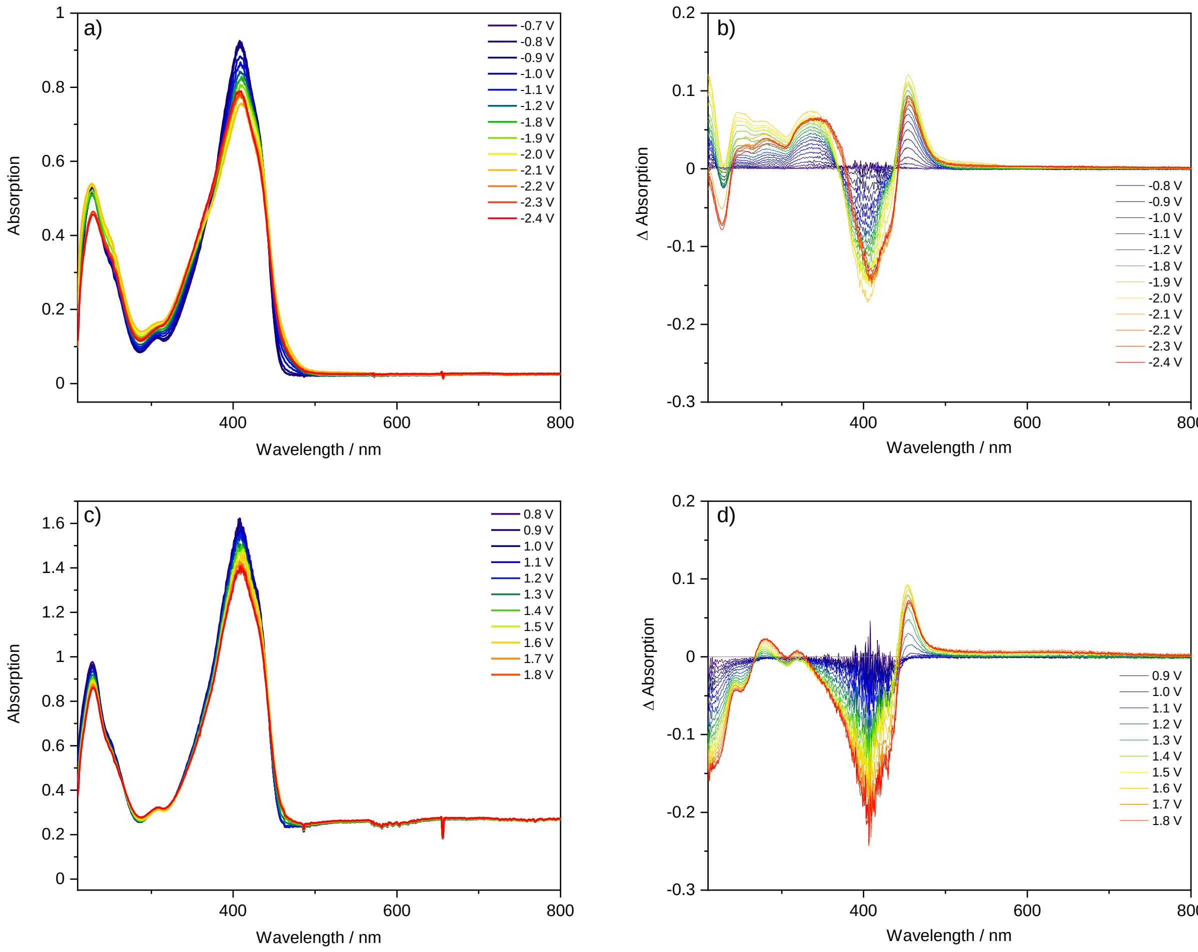

Figure S11: UV-vis SEC of **PS**. a) Reduction. b) differential spectrum of reduction. c) Oxidation. d) differential spectrum of oxidation.

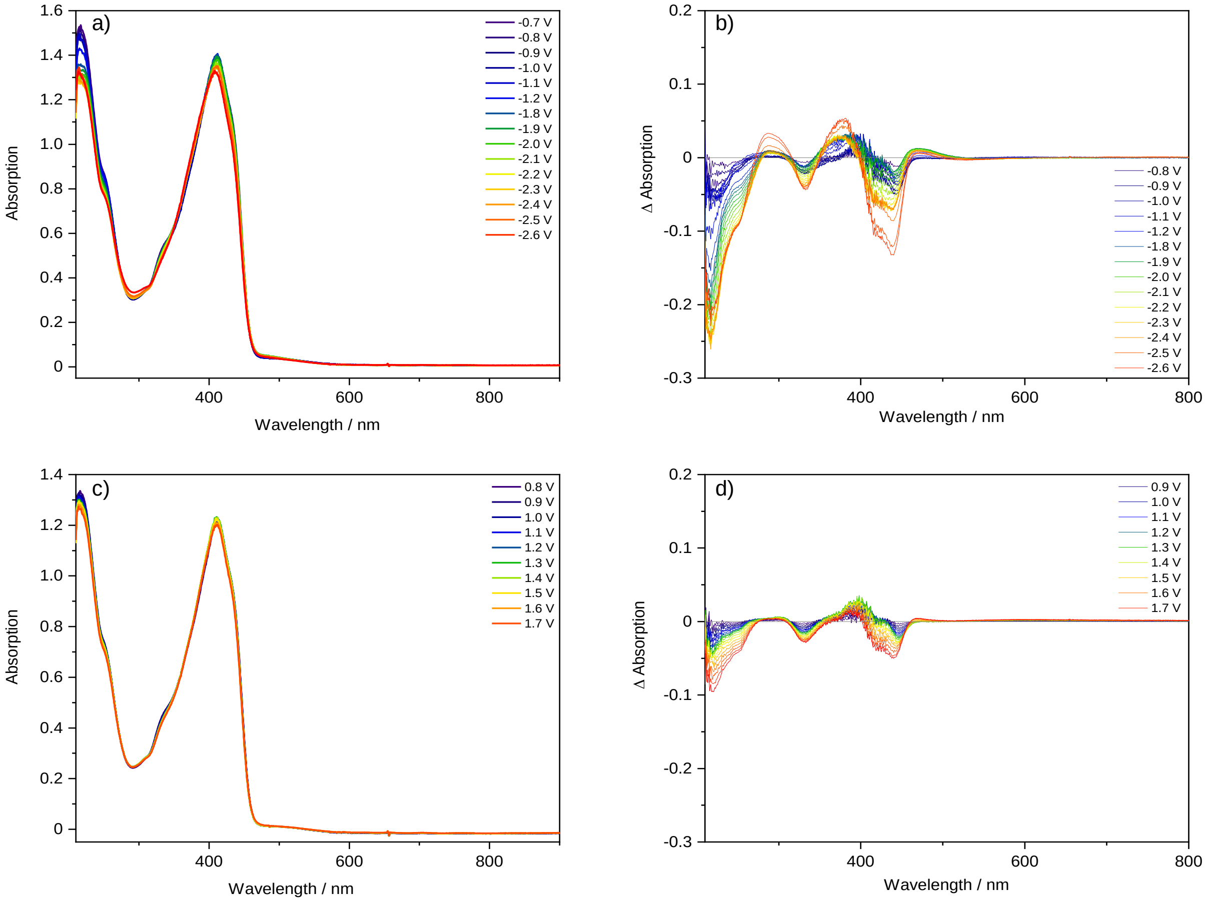

Figure S12: UV-vis SEC of **PS-CAT**. a) Reduction. b) differential spectrum of reduction. c) Oxidation. d) differential spectrum of oxidation.

Computational details

All quantum chemical calculations determining structural and electronic properties of **PS-CAT** were performed using the Gaussian 16 program.[17] To reduce the computational demand without affecting the photophysical properties of the present photocatalyst, the terminal alkyl groups of the thiophene-based chromophore were approximated by methyl groups. The fully relaxed equilibrium geometry of **PS-CAT** was obtained within the singlet ground state at the density functional level of theory (DFT) by means of the B3LYP[18] XC functional. The def2-SVP[18] basis set was applied for all atoms. A vibrational analysis was carried out to verify that a minimum on the potential energy (hyper)surface (PES) was obtained. To correct for the lack of anharmonicity and the approximate treatment of electron correlation, the harmonic frequencies were scaled by the factor 0.97.[19]

Subsequently, excited state properties such as excitation energies, oscillator strengths and electronic characters were calculated within the Frack-Condon (FC) structure at the time-dependent DFT (TDDFT) level of theory. Therefore, the 100 lowest singlet and the 100 lowest triplet excited states were calculated, while the same XC functional and basis set were applied as for the preceding ground state calculations. Several computational as well as joint spectroscopic-theoretical studies on structurally related [FeFe]-hydrogenase mimics showed that this computational protocol enables an accurate prediction of ground and excited states properties with respect to experimental data, *e.g.* structural and electrochemical properties as well as with respect to UV-vis absorption.[20] In particular, a reliable description of the singlet-triplet splitting is of uttermost importance to elucidate excited state relaxation processes for hydrogenase mimics. State-of-the-art *ab initio* simulations, *e.g.* via CASPT2, RASPT2 and CCSD(T), show that B3LYP is able to predict such singlet-triplet splitting with reasonable accuracy for structurally related hydrogenase mimics,[21] while protonation of the active site may hamper an accurate description at the B3LYP level of theory.[22] Such protonated species were not investigated in the present study. Several theoretical studies addressing the photophysics of the active site of such [FeFe]-hydrogenase models point to a dependency of the amount of exact exchange on the excited state properties of interest. However, in case of transition metal complexes, *e.g.* **PS-CAT**,[20a, 23] a balanced description – as provided by the present computational setup – is essential that allows to investigate the excited state properties of the photosensitzer, of the catalytic center as well as the intramolecular interactions among both moieties.[24] The extraordinary rich photophysics and photochemistry of such transition metal complexes originates from the manifold of excited states involved, *i.e.*, metal-to-ligand charge transfer (MLCT), ligand-to-metal charge transfer (LMCT), ligand-to-ligand charge transfer (LLCT), intra-ligand charge transfer (ILCT), intra-ligand (IL) and metal-centered (MC) character. The present computational protocol, as provided by the present computational setup, is essential to assess the photophysics of transition metal complexes. Effects of interaction with a solvent (tetrahydrofuran, THF: $\varepsilon = 7.4257$, $n = 1.407$) were taken into account on the ground and excited states properties by the solute electron density (SMD) variant of the integral equation formalism of the polarizable continuum model.[25] The non-equilibrium procedure of solvation was used for the calculation of the excitation energies within the Franck-Condon point, which is well adapted for processes where only the fast reorganization of the electronic distribution of the solvent is important. All calculations were performed including D3 dispersion correction with Becke-Johnson damping.[26]

Furthermore, excited state relaxation pathways associated to photo-induced electron transfer as well as energy transfer were studied. Therefore, fully relaxed equilibrium structures were obtained at the DFT and TDDFT for prominent triplet states, involved in the electron and energy transfer channels. Starting from the FC point, the triplet ground state (T_1) of **PS-CAT** was optimized at the DFT level of theory. This metal-centered (^{3}MC) state features one unpaired electron in the σ_{FeFe} orbital and one unpaired electron in the σ_{FeFe^*} orbital. Thus, the Fe-Fe bond order is decreased from one to zero upon population transfer from S_0 to T_1. This decreased bond order is well reflected

by the increased Fe-Fe distance from 2.503 (FC point) to 3.137 Å (^{3}MC equilibrium structure). Furthermore, the locally excited $^3\pi\pi^*$ state of the thiophene-based chromophore, T_2, as well as the triplet charge-separated (^{3}CS) were optimized at the TDDFT level of theory using pysisyphus[27] – our lately introduced external optimizer which is also aware of excited states. Structural relaxation within both states leads to a planarization of the in the FC point slightly twisted chromophore, see Table S4. As expected, the Fe-Fe bond is unaffected by the equilibration of the $^3\pi\pi^*$ state (d_{FeFe} = 2.501 Å), while the LMCT from the chromophore to the active site of the $[Fe_2S_2]$-cluster (^{3}CS state) leads to an elongation of the Fe-Fe to d_{FeFe} = 2.753 Å. This elongation is a result of the decreased bond order from one (S_0) to 0.5 upon electron transfer into the σ_{FeFe}^* orbital. The equilibrium procedure of solvation was used for all ground and excited state optimizations. Finally, the redox potential of the ^{3}CS state (T_2, see Table S7 $\Delta\Delta E$ = -0.45 eV) was obtained based on its equilibrium energy and the ground state energy of the singly reduced **PS-CAT** (D_0) with respect to the Fc^+/Fc redox couple – obtained at the same level of theory.

Table S4: Selected structural properties such as bond lengths (d_{FeFe}), bond angles (α_i) and dihedral angels (δ_i) of **PS-CAT** within its fully optimized equilibrium structures, *i.e.* at the Franck-Condon (FC) point, the locally excited intraligand triplet state ($^3\pi\pi^*$), the triplet charge-separated state (^{3}CS) and the triplet metal-centered (^{3}MC).

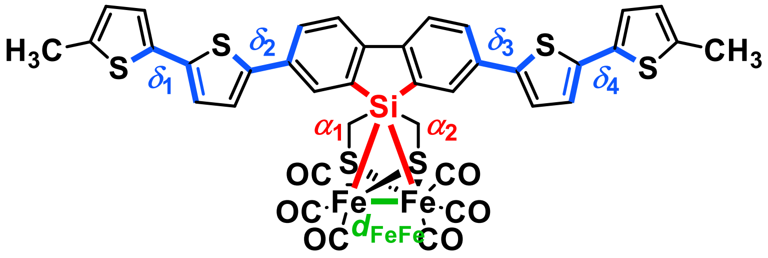

Geometry	d_{FeFe} / Å	α_1 / °	α_2 / °	δ_1 / °	δ_2 / °	δ_3 / °	δ_4 / °
FC	2.5030	110.7	123.0	11.5	-14.7	-18.0	9.0
$^3\pi\pi^*$	2.5013	110.3	123.5	5.6	-7.9	-0.3	-0.1
^{3}CS	2.7530	109.4	120.9	0.1	-2.8	0.3	0.7
^{3}MC	3.1366	91.2	130.1	10. 6	-6.4	-15.1	5.6

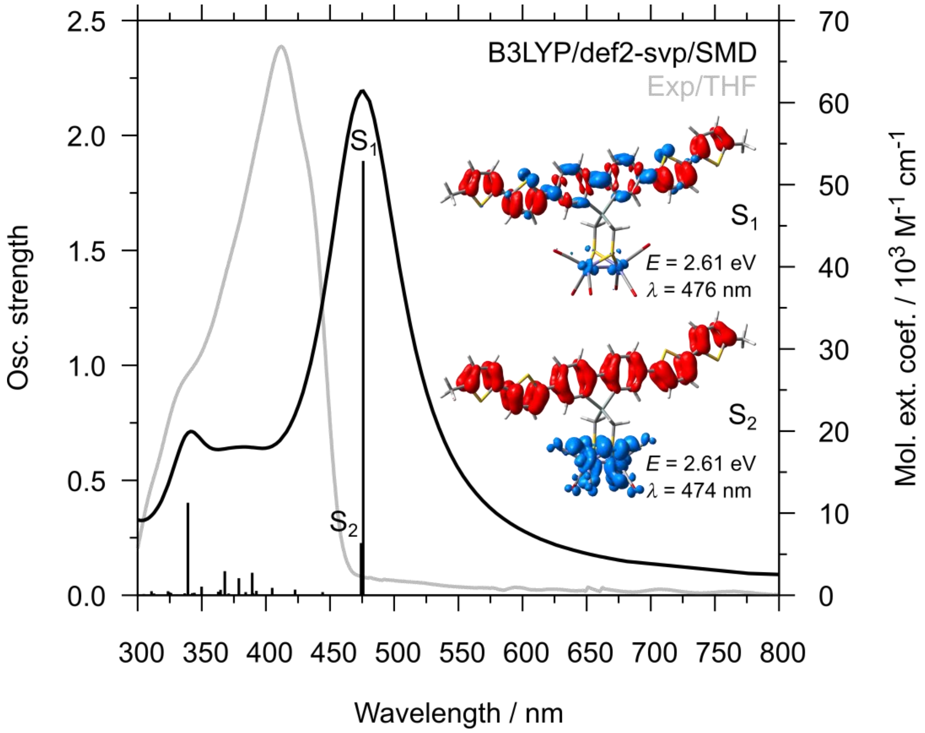

Figure S13: Experimental (grey) and simulated (black) UV-vis absorption spectrum of **PS-CAT** in THF. The nature of electronic transitions (into S_1 and S_2) is illustrated by charge density differences (CDDs); charge transfer takes place from red to blue.

Table S5: Excitation energies (Δ*E*), excitation wavelengths (*λ*), oscillator strengths (*f*) and electronic character of spin-allowed singlet-singlet and spin-forbidden singlet-triplet excitations within the equilibrium structure of the singlet ground state (S_0, Franck-Condon (FC) point) as well as spin-allowed doublet-doublet excitations within the equilibrium structure of the doublet ground state of the singly reduced species. Experimental excitation wavelengths (λ_{exp}) are assigned.

Spin-allowed $S_0 \rightarrow S_i$ excitations (FC point)				
Excitation (Character)	Δ*E* / eV	*λ* / nm	*f*	λ_{exp} / nm
S_1 (IL)	2.61	476	1.8895	412
S_2 (LMCT)	2.61	474	0.2273	412
Spin-forbidden $S_0 \rightarrow T_i$ excitations (FC point)				
Excitation (Character)	Δ*E* / eV	*λ* / nm	*f*	λ_{exp} / nm
T_1 (MC)	1.07	1155	-	-
T_2 (IL)	1.82	681	-	-
T_{10} (LMCT)	2.62	474	-	-
Spin-allowed $D_0 \rightarrow D_i$ excitations (singly reduced D_0 equilibrium structure)				
D_4 (MC)	1.76	704	0.0721	580
D_{25} (IL)	2.64	469	2.0524	399

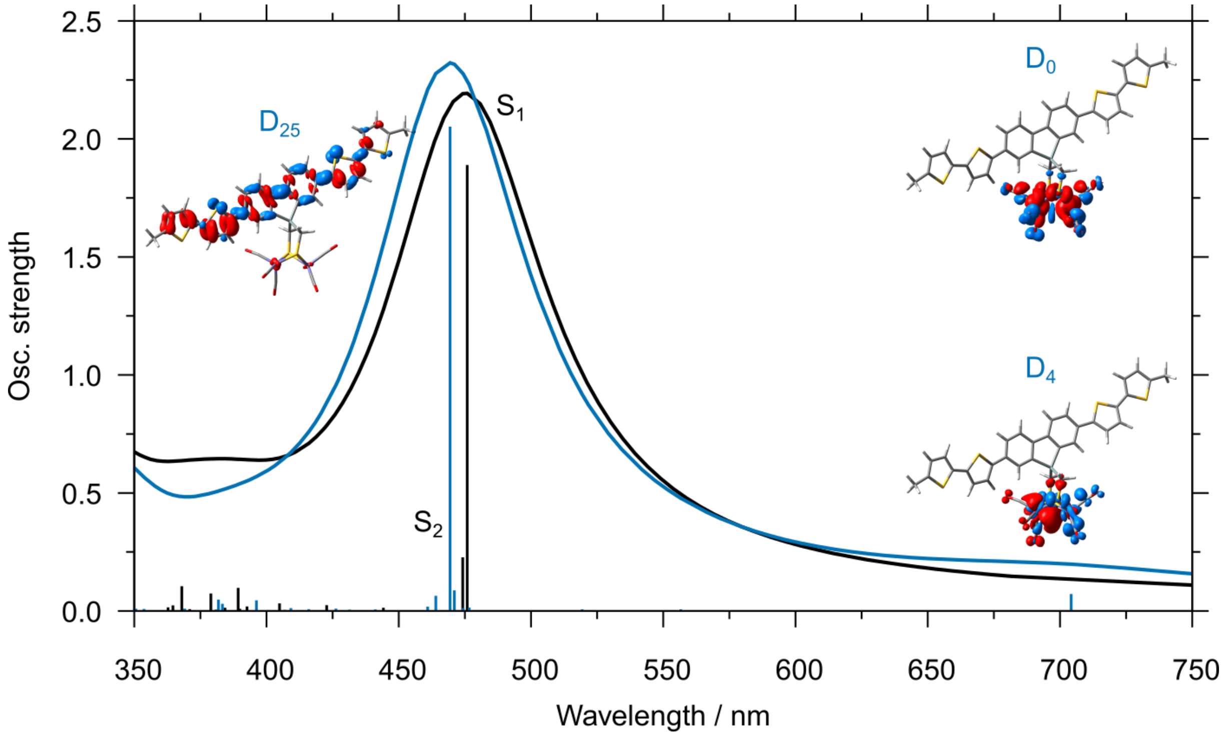

Figure S14: Simulated UV-vis absorption spectrum of the non-reduced singlet (black) and the singly reduced doublet (blue) species of **PS-CAT** in THF. The spin density of the doublet ground state (D_0) show that single reduction occurs at the [FeFe]-center, *i.e.* population of the σ_{FeFe}^* molecular orbital. The nature of the $D_0 \rightarrow D_{25}$ transitions is illustrated by a charge density difference (CDD); charge transfer takes place from red to blue.

Table S6: Vibrational normal modes associated to the vibrational structure in the electronic absorption spectrum of **PS-CAT** in Figure 3a). All vibrational modes (modes 162 to 165) in vicinity of the measured vibrational feature (~1250 cm^{-1}) correspond to CC stretching and CH bending accounting for the altered electronic structure of the aromatic system upon ππ* excitation. All frequencies were scaled by a factor for 0.97.

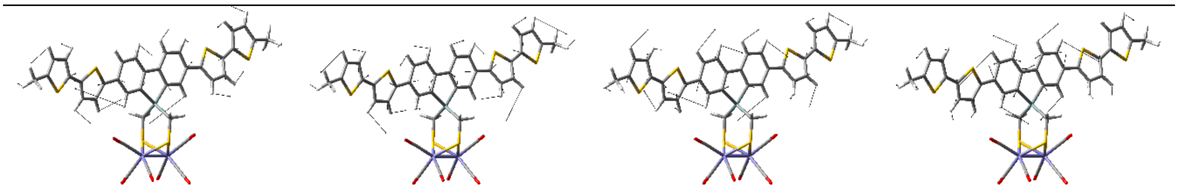

mode 162 (1243 cm^{-1})	mode 163 (1249 cm^{-1})	mode 164 (1253 cm^{-1})	mode 165 (1264 cm^{-1})

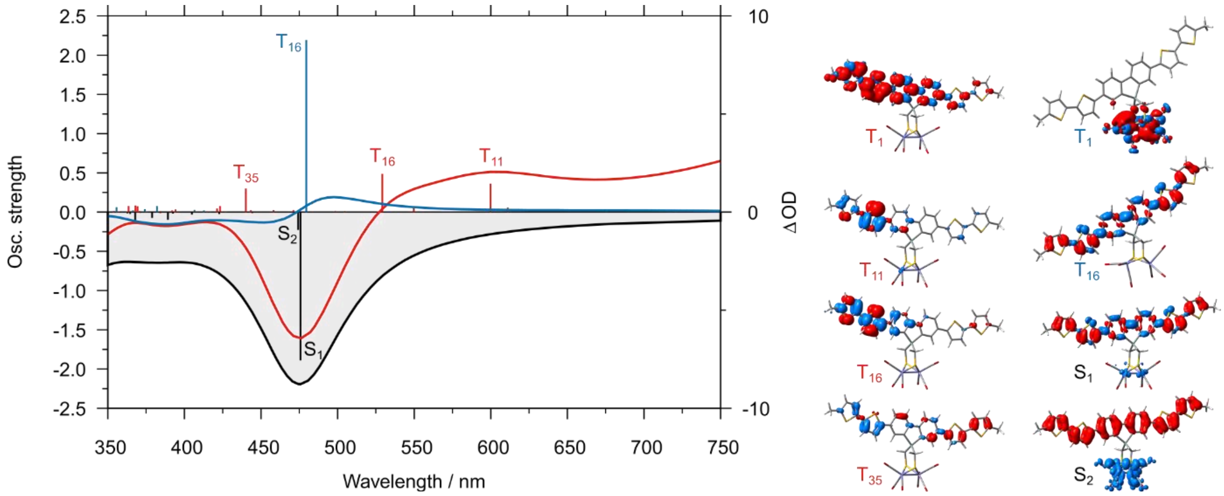

Figure S15: Simulated transient absorption spectra of **PS-CAT**. Positive signals (excited state absorption) originate from spin-allowed triplet-triplet excitations within the equilibrium structures of $^3\pi\pi^*$ (red) and ^{3}MC (blue), respectively. Negative signals (ground state bleach) stem from spin-allowed singlet-singlet excitations (black) within the equilibrium structure of the singlet ground state. Excitations of interest are labelled in red and blue accordingly. Spin densities of the triplet ground states (T_1) obtained within $^3\pi\pi^*$ (red) and ^{3}MC (blue) and charge density differences (CDDs) illustrating the respective electronic transitions are shown; charge transfer takes place from red to blue.

Table S7: Relative energies and charge density differences of all states involved in the proposed excited state relaxation scheme, see Figure 3. Energies are given with respect to the singlet ground state (S_0) in the Franck-Condon point (FC, S_0 equilibrium structure). Charge transfer takes place from red to blue.

	FC	
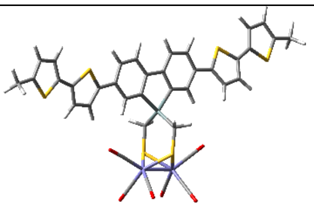 S_0 (0.00 eV)	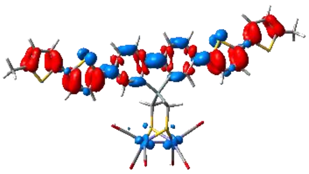 S_1 ($^1\pi\pi^*$, 2.61 eV)	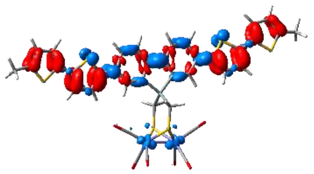 S_2 (^{1}CS, 2.61 eV)
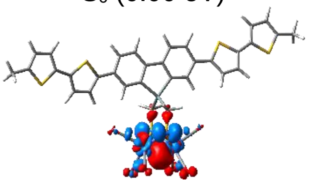 T_1 (^{3}MC, 1.07 eV)	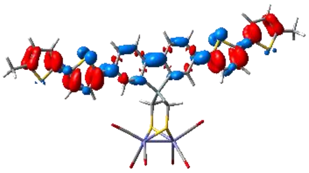 T_2 ($^3\pi\pi^*$, 1.82 eV)	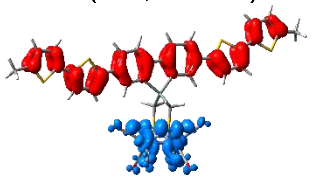 T_{10} (^{3}CS, 2.62 eV)
	$^3\pi\pi^*$	
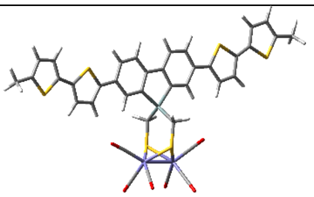 S_0 (0.36 eV)	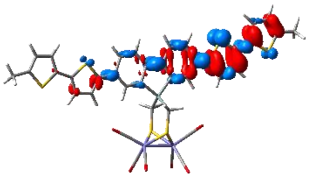 S_1 ($^1\pi\pi^*$, 2.51 eV)	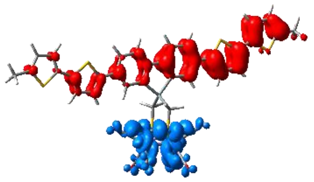 S_2 (^{1}CS, 2.67 eV)
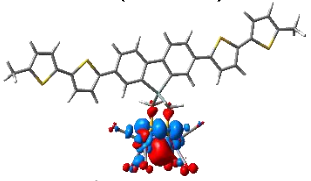 T_1 (^{3}MC, 1.44 eV)	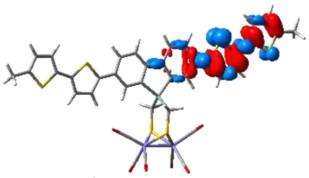 T_2 ($^3\pi\pi^*$, 1.49 eV)	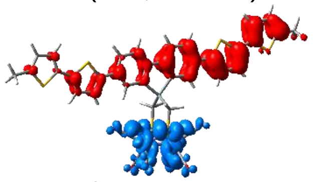 T_5 (^{3}CS*, 2.67 eV)
	^{3}CS	
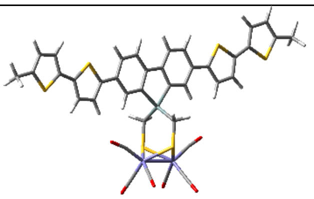 S_0 (0.53 eV)	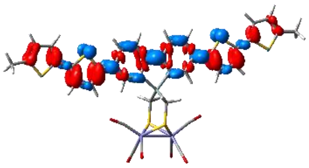 S_4 ($^1\pi\pi^*$, 2.84 eV)	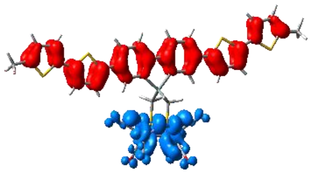 S_1 (^{1}CS, 2.06 eV)
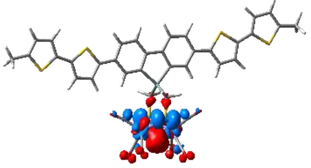 T_1 (^{3}MC, -0.12 eV)	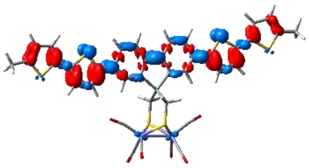 T_2 ($^3\pi\pi^*$, 2.03 eV)	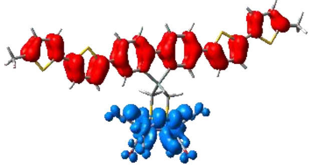 T_3 (^{3}CS*, 2.06 eV)
	^{3}MC	
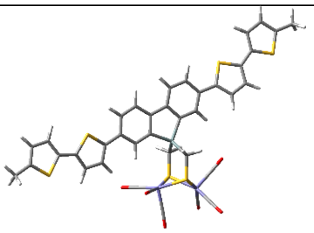 S_0 (1.25 eV)	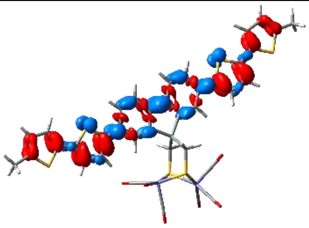 S_{14} ($^1\pi\pi^*$, 2.84 eV)	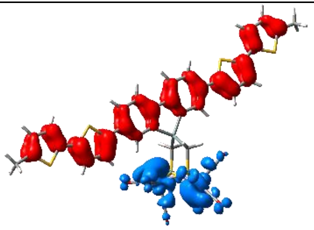 S_2 (^{1}CS, 2.73 eV)

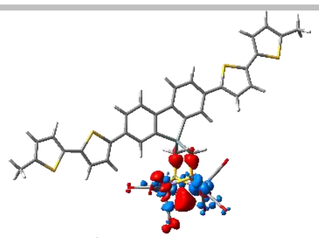

T_1 (^{3}MC, -0.06 eV)

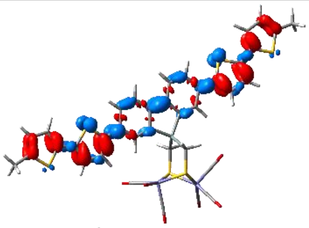

T_{10} ($^3\pi\pi^*$, 3.04 eV)

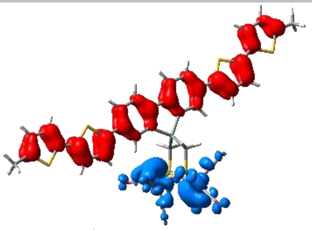

T_5 (^{3}CS*, 2.73 eV)

References

[1] J.-C. Chen, Y.-C. Liu, J.-J. Ju, C.-J. Chiang, Y.-T. Chern, *Polymer* **2011**, *52*, 954-964.

[2] a) S. D. Oosterhout, V. Savikhin, J. Zhang, Y. Zhang, M. A. Burgers, S. R. Marder, G. C. Bazan, M. F. Toney, *Chem. Mater.* **2017**, *29*, 3062-3069; b) S. A. Ponomarenko, E. A. Tatarinova, A. M. Muzafarov, S. Kirchmeyer, L. Brassat, A. Mourran, M. Moeller, S. Setayesh, D. de Leeuw, *Chem. Mater.* **2006**, *18*, 4101-4108.

[3] a) V. I. Handmann, R. Bertermann, C. Burschka, R. Tacke, *J. Organomet. Chem.* **2000**, *613*, 19-25; b) J. O. Daiss, K. A. Barth, C. Burschka, P. Hey, R. Ilg, K. Klemm, I. Richter, S. A. Wagner, R. Tacke, *Organometallics* **2004**, *23*, 5193-5197.

[4] D. Hong, Y. Tsukakoshi, H. Kotani, T. Ishizuka, T. Kojima, *J. Am. Chem. Soc.* **2017**, *139*, 6538-6541.

[5] G. M. Sheldrick, *Acta Cryst. C* **2015**, *71*, 3-8.

[6] S. Stoll, A. Schweiger, *J. Magn. Reson.* **2006**, *178*, 42-55.

[7] C. Gu, D. Zhu, M. Qiu, L. Han, S. Wen, Y. Li, R. Yang, *New J. Chem.* **2016**, *40*, 7787-7794.

[8] R. Goy, L. Bertini, T. Rudolph, S. Lin, M. Schulz, G. Zampella, B. Dietzek, F. H. Schacher, L. De Gioia, K. Sakai, W. Weigand, *Chem. - Eur. J.* **2017**, *23*, 334-345.

[9] U.-P. Apfel, C. R. Kowol, E. Morera, H. Görls, G. Lucente, B. K. Keppler, W. Weigand, *Eur. J. Inorg. Chem.* **2010**, *2010*, 5079-5086.

[10] R. Goy, U.-P. Apfel, C. Elleouet, D. Escudero, M. Elstner, H. Görls, J. Talarmin, P. Schollhammer, L. Gonzalez, W. Weigand, *Eur. J. Inorg. Chem.* **2013**, *2013*, 4466-4472.

[11] Y. Tamaki, K. Koike, T. Morimoto, O. Ishitani, *J. Catal.* **2013**, *304*, 22-28.

[12] Y. Pellegrin, F. Odobel, *C. R. Chim.* **2017**, *20*, 283-295.

[13] V. V. Pavlishchuk, A. W. Addison, *Inorg. Chim. Acta* **2000**, *298*, 97-102.

[14] a) R. Goy, L. Bertini, H. Görls, L. De Gioia, J. Talarmin, G. Zampella, P. Schollhammer, W. Weigand, *Chem. - Eur. J.* **2015**, *21*, 5061-5073; b) R. Goy, L. Bertini, T. Rudolph, S. Lin, M. Schulz, G. Zampella, B. Dietzek, F. H. Schacher, L. De Gioia, K. Sakai, W. Weigand, *Chem. - Eur. J.* **2017**, *23*, 334-345.

[15] S. J. Borg, T. Behrsing, S. P. Best, M. Razavet, X. Liu, C. J. Pickett, *J. Am. Chem. Soc.* **2004**, *126*, 16988-16999.

[16] M. Mirmohades, S. Pullen, M. Stein, S. Maji, S. Ott, L. Hammarström, R. Lomoth, *J. Am. Chem. Soc.* **2014**, *136*, 17366-17369.

[17] M. J. Frisch, G. W. Trucks, H. B. Schlegel, G. E. Scuseria, M. A. Robb, J. R. Cheeseman, G. Scalmani, V. Barone, G. A. Petersson, H. Nakatsuji, *et al.*, in *Gaussian 16 Rev. B.01*, Wallingford, CT, **2016**.

[18] a) F. Weigend, R. Ahlrichs, *Phys. Chem. Chem. Phys.* **2005**, *7*, 3297-3305; b) F. Weigend, *Phys. Chem. Chem. Phys.* **2006**, *8*, 1057-1065.

[19] J. P. Merrick, D. Moran, L. Radom, *J. Phys. Chem. A* **2007**, *111*, 11683-11700.

[20] a) P. E. M. Siegbahn, R.-Z. Liao, *J. Phys. Chem. A* **2020**, *124*, 10540-10549; b) R. Goy, L. Bertini, C. Elleouet, H. Görls, G. Zampella, J. Talarmin, L. De Gioia, P. Schollhammer, U.-P. Apfel, W. Weigand, *Dalton Trans.* **2015**, *44*, 1690-1699; c) P. Buday, P. Seeber, C. Zens, H. Abul-Futouh, H. Görls, S. Graefe, P. Matczak, S. Kupfer, W. Weigand, G. Mloston, *Chem. - Eur. J.* **2020**, *26*, 11412-11416; d) L. Bertini, C. Greco, L. De Gioia, P. Fantucci, *J. Phys. Chem. A* **2009**, *113*, 5657-5670; e) H. Abul-Futouh, Y. Zagranyarski, C. Müller, M. Schulz, S. Kupfer, H. Görls, M. El-khateeb, S. Gräfe, B. Dietzek, K. Peneva, W. Weigand, *Dalton Trans.* **2017**, *46*, 11180-11191.

[21] a) M. G. Delcey, K. Pierloot, Q. M. Phung, S. Vancoillie, R. Lindh, U. Ryde, *Phys. Chem. Chem. Phys.* **2014**, *16*, 7927-7938; b) P. E. M. Siegbahn, R.-Z. Liao, *ACS Catal.* **2020**, *10*, 5603-5613.

[22] a) G. Dong, U. Ryde, *J. Biol. Inorg. Chem.* **2016**, *21*, 383-394; b) G. Dong, Q. M. Phung, S. D. Hallaert, K. Pierloot, U. Ryde, *Phys. Chem. Chem. Phys.* **2017**, *19*, 10590-10601; c) S. Qiu, Q. Li, Y. Xu, S. Shen, C. Sun, *Wiley Interdiscip. Rev. Comput. Mol. Sci.* **2020**, *10*, e1422.

[23] a) J. L. Bingaman, C. L. Kohnhorst, G. A. Van Meter, B. A. McElroy, E. A. Rakowski, B. W. Caplins, T. A. Gutowski, C. J. Stromberg, C. E. Webster, E. J. Heilweil, *J. Phys. Chem. A* **2012**, *116*, 7261-7271; b) M. Sensi, C. Baffert, L. Fradale, C. Gauquelin, P. Soucaille, I. Meynial-Salles, H. Bottin, L. de Gioia, M. Bruschi, V. Fourmond, C. Léger, L. Bertini, *ACS Catal.* **2017**, *7*, 7378-7387.

[24] a) D. Escudero, in *Transition Metals in Coordination Environments: Computational Chemistry and Catalysis Viewpoints* (Eds.: E. Broclawik, T. Borowski, M. Radoń), Springer International Publishing, Cham, **2019**, pp. 259-287; b) L. González, D. Escudero, L. Serrano-Andrés, *ChemPhysChem* **2012**, *13*, 28-51; c) C. Latouche, D. Skouteris, F. Palazzetti, V. Barone, *J. Chem. Theory Comput.* **2015**, *11*, 3281-3289; d) G. E. Shillito, T. B. J. Hall, D. Preston, P. Traber, L. Wu, K. E. A. Reynolds, R. Horvath, X. Z. Sun, N. T. Lucas, J. D. Crowley, M. W. George, S. Kupfer, K. C. Gordon, *J. Am. Chem. Soc.* **2018**, *140*, 4534-4542; e) L. Zedler, A. K. Mengele, K. M. Ziems, Y. Zhang, M. Wächtler, S. Gräfe, T. Pascher, S. Rau, S. Kupfer, B. Dietzek, *Angew. Chem. Int. Ed.* **2019**, *58*, 13140-13148.

[25] a) A. V. Marenich, C. J. Cramer, D. G. Truhlar, *J. Chem. Phys. B* **2009**, *113*, 6378-6396; b) B. Mennucci, C. Cappelli, C. A. Guido, R. Cammi, J. Tomasi, *J. Phys. Chem. A* **2009**, *113*, 3009-3020.

[26] S. Grimme, S. Ehrlich, L. Goerigk, *J. Comput. Chem.* **2011**, *32*, 1456-1465.

[27] J. Steinmetzer, S. Kupfer, S. Gräfe, *Int. J. Quantum Chem.* **2021**, *121*, e26390.

Author Contributions

Philipp Buday (synthesis, photocatalysis experiments, writing of original draft, equal contribution), Chizuru Kasahara (synthesis, equal contribution), Elisabeth Hofmeister (EPR and SEC experiments), Daniel Kowalczyk (development 3D photoreactor platform), Micheal K. Farh (synthesis, supporting), Saskia Riediger (synthesis, supporting), Martin Schulz (irradiation experiments, supporting), Maria Wächtler (photophysical experiments), Shunsuke Furukawa (co-supervision), Masaichi Saito (supervision), Dirk Ziegenbalg (development 3D photoreactor platform), Stefanie Gräfe (quantum chemical simulation), Peter Bäuerle (supervision, conceptualization), Stephan Kupfer (quantum chemical simulation, lead), Benjamin Dietzek-Ivanšić (supervision, photophysical experiments, lead), and Wolfgang Weigand (supervision, conceptualization, writing manuscript, lead)
